# A novel anoikis-related gene prognostic signature and its correlation with the immune microenvironment in colorectal cancer

**DOI:** 10.3389/fgene.2023.1186862

**Published:** 2023-05-30

**Authors:** Yu Xiao, Han Zhou, Yiran Chen, Libin Liu, Qian Wu, Hui Li, Peicheng Lin, Jinluan Li, Junxin Wu, Lirui Tang

**Affiliations:** Department of Radiation Oncology, Clinical Oncology School of Fujian Medical University, Fujian Cancer Hospital, Fuzhou, China

**Keywords:** colorectal cancer, anoikis, tumor microenvironment, immunotherapy, prognosis

## Abstract

**Background:** Anoikis is a type of apoptosis associated with cell detachment. Resistance to anoikis is a focal point of tumor metastasis. This study aimed to explore the relationship among anoikis-related genes (ARGs), immune infiltration, and prognosis in colorectal cancer (CRC).

**Methods:** The transcriptome profile and clinical data on patients with CRC were retrieved from The Cancer Genome Atlas and Gene Expression Omnibus databases. Patients were divided into two clusters based on the expression of ARGs. Differences between the two ARG molecular subtypes were analyzed in terms of prognosis, functional enrichment, gene mutation frequency, and immune cell infiltration. An ARG-related prognostic signature for predicting overall survival in patients with CRC was developed and validated using absolute value convergence and selection operator (LASSO) regression analysis. The correlation between the signature risk score and clinicopathological features, immune cell infiltration, immune typing, and immunotherapy response was analyzed. The risk score combined with clinicopathological characteristics was used to construct a nomogram to assess CRC patients’ prognosis.

**Results:** Overall, 151 ARGs were differentially expressed in CRC. Two ARG subtypes, namely, ARG-high and ARG-low groups, were identified and correlated with CRC prognosis. The gene mutation frequency and immune, stromal, and ESTIMATE scores of the ARG-high group were higher than those of the ARG-low group. Moreover, CD8, natural killer cells, M1 macrophages, human leukocyte antigen (HLA), and immune checkpoint-related genes were significantly increased in the ARG-high group. An optimized 25-gene CRC prognostic signature was successfully constructed, and its prognostic predictive ability was validated. The high-risk score was correlated with T, N, M, and TNM stages. Risk scores were negatively correlated with dendritic cells, eosinophils, and CD4 cells, and significantly positively correlated with regulatory T cells. Patients in the high-risk group were more likely to exhibit immune unresponsiveness. Finally, the nomogram model was constructed and showed good prognostic predictive power.

**Conclusion:** ARGs are associated with clinicopathological features and the prognosis of CRC, and play important roles in the immune microenvironment. Herein, we underpinned the usefulness of ARGs in CRC to develop more effective immunotherapy techniques.

## 1 Introduction

Colorectal cancer (CRC) is one of the most common malignant tumors worldwide, with high recurrence and mortality rates that seriously threaten human health. In 2020, more than 1.9 million new CRC cases and 935,000 deaths were reported (Sung et al., 2021). Despite recent advances in multimodal methods, such as surgery, chemotherapy, and radiation, distant metastasis, recurrence, and death rates for CRC remain high (Yamamoto et al., 2021). Considering the limitations to the CRC treatment, novel therapeutic strategies are needed to improve clinical outcomes. Therefore, reliable prognostic signatures are urgently needed to develop more practical therapies.

Cells undergo a unique type of apoptosis termed anoikis as they detach from the extracellular matrix or are defective in cell adhesion (Taddei et al., 2012). Initially, anoikis was regarded as a natural physiological process for epithelial and endothelial cells to maintain regular developmental homeostasis by preventing the reattachment of detached cells to the new extracellular matrix and arresting their aberrant growth, which is an essential defense mechanism for maintaining cellular stability in the organism (Frisch and Francis, 1994; Paoli et al., 2013). Tumor cells rapidly exhibit multiple mechanisms to resist anoikis under the pressure of natural selection, while dysregulated anoikis is a specific hallmark of abnormal tumor cell proliferation and the formation of metastatic foci in distant organs (Frisch and Screaton, 2001; Chiarugi and Giannoni, 2008). Tumor cells can switch the expression pattern of integrins by adapting the extracellular matrix to the metastatic area, affecting the downstream signaling cascade to enhance cell survival and prevent programmed cell death (Morozevich et al., 2003). Epithelial–mesenchymal transition is a key event in tumor progression and the acquisition of drug resistance. Critical genes in the epithelial–mesenchymal transition process activate specific pro-survival signals and play a key role in escaping anoikis (Buchheit et al., 2014). Another mechanism used by tumor cells to escape anoikis is the use of intrinsic or environmental factors that lead to constitutive activation of pathways affecting cell survival (e.g., Src family kinases and the PI3K-Akt pathway) (Boyer et al., 1997; Khwaja et al., 1997). Oncogenic Ras interacts with Bcl-2 family proteins, causing downregulation of the pro-apoptotic protein Bak and, thus, resistance to anoikis (Cheng et al., 2001).

Currently, anoikis resistance correlates with a variety of characteristic tumor processes. Du et al. revealed biological interactions between critical elements of anoikis resistance and angiogenesis, which promoted peritoneal metastasis in gastric cancer and showed promise as a potential prognostic biomarker and therapeutic target (Arif et al., 2021). Bárbara et al. demonstrated that metabolic remodeling and antioxidant processes in breast cancer can promote anoikis resistance, thus promoting the survival of breast cancer cells at circulating and metastatic sites (Sousa et al., 2020). However, the relevance of anoikis resistance to immune escape and its potential as a biomarker for prognosis and immunotherapy response in patients with CRC still requires further exploration.

This study comprehensively evaluated the expression profile of anoikis-related genes (ARGs) and used two calculation algorithms, namely, CIBERSORT (Cell-type Identification by Estimating Relative Subsets of RNA Transcripts) and ESTIMATE (Estimation of STromal and Immune cells in MAlignant Tumor tissues using Expression data), to provide a comprehensive overview of the immune microenvironment within the tumor. First, patients with CRC were classified into two ARG subtypes, according to the expression levels of ARGs. To further explore the biological properties of the two ARG subtypes, we analyzed survival, functional enrichment, and immune infiltration between the two ARG subtypes. Moreover, based on the expression of ARGs, we established a risk prognostic signature to predict CRC overall survival (OS) and analyzed the immune infiltration status of CRC. Our results may contribute to additional reference information on prognostic biomarkers and molecular mechanisms of anoikis in CRC.

## 2 Materials and methods

### 2.1 Data collection

**FIGURE 1:**
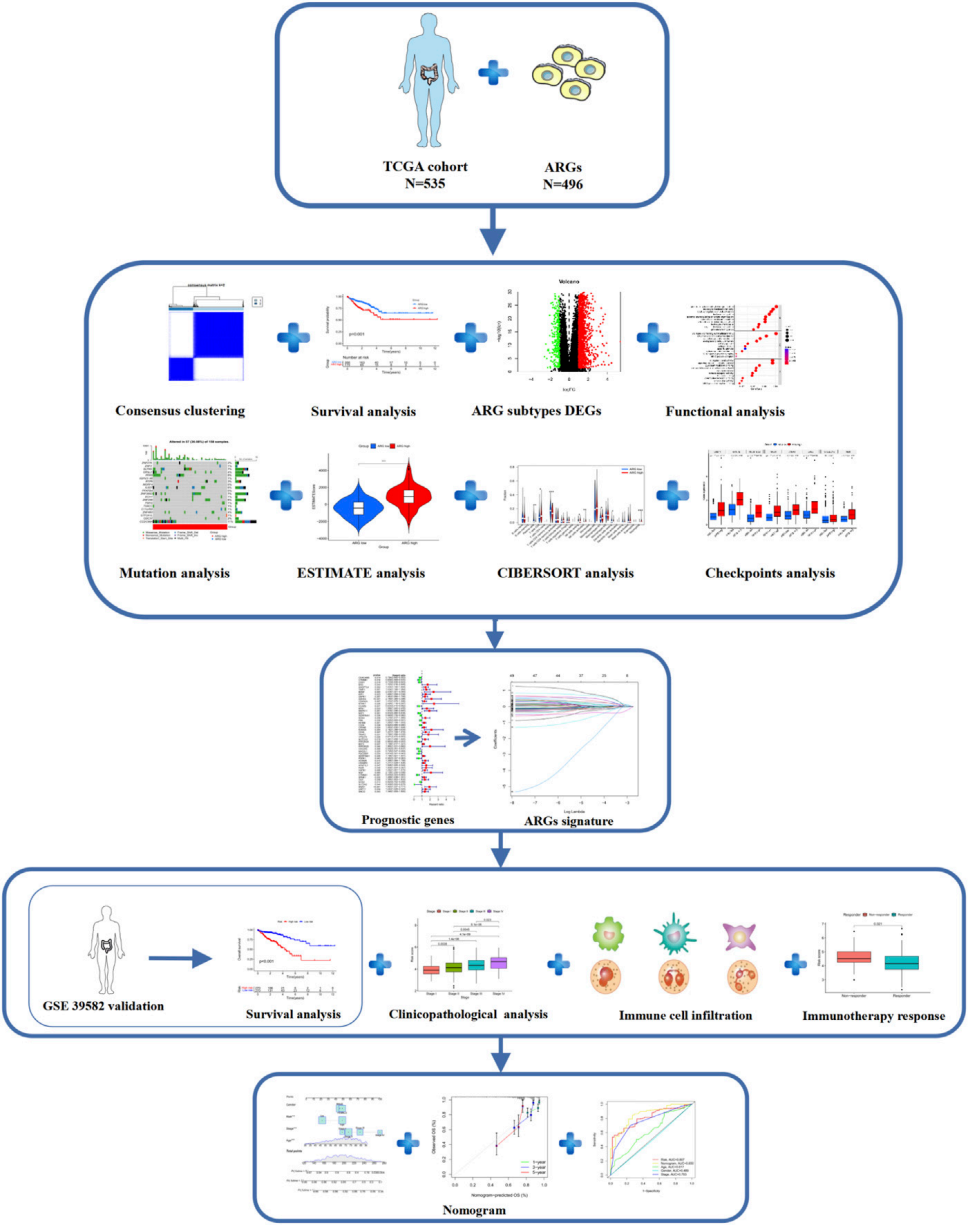
Flowchart of this research.

The study flow is illustrated in Figure 1. CRC clinical and mRNA expression data were downloaded from The Cancer Genome Atlas (TCGA) (https://portal.gdc.cancer.gov/) and Gene Expression Omnibus (GEO) (https://www.ncbi.nlm.nih.gov/geo/) databases’ GSE 39582 dataset. The transcriptome data in the TCGA COAD/READ dataset include 568 tumor samples and 44 normal tissue samples, corresponding to 540 complete clinicopathological data points (Supplementary Table S1). The GSE39582 dataset contains 585 CRC transcriptome and clinicopathological data points (Supplementary Table S2).

### 2.2 Identification of differential genes

We extracted 496 ARGs from GeneCards (https://www.genecards.org/) (Supplementary Table S3). The “limma” package was used to distinguish differentially expressed genes (DEGs) in CRC with *p*-values <0.05.

### 2.3 ARG subtype identification

To categorize patients into discrete molecular subgroups based on ARG expression, the R package “ConsensusClusterPlus” was used for unsupervised consensus clustering analysis. Clustering boosted the intragroup correlation while decreasing the intergroup correlation. The “limma” package in R was used to find DEGs among different ARG subtypes. A fold-change of 1 and *p* < 0.05 were set for selected DEGs. To further investigate the probable activities of DEGs associated with ARG subtypes, Gene Ontology (GO), Kyoto Encyclopedia of Genes and Genomes (KEGG), and Gene Set Enrichment Analysis (GSEA) were used to analyze. The “maftools” package was used to analyze gene mutations of the two subtypes. Computational tools, ESTIMATE and CIBERSORT, were used to evaluate the immune microenvironment and infiltration of immune cells. Furthermore, we used the “limma” package to analyze the differences in the expression of HLA-related genes and immune checkpoint-related genes between the two ARG subtypes.

### 2.4 Development and validation of the ARG prognostic signature

Univariate Cox regression analysis was used to identify DEGs associated with OS prognosis in CRC. TCGA cohort COAD/READ dataset was used as the training set. The GEO cohort GSE39582 dataset was used as the testing set. The least absolute shrinkage and selection operator (LASSO) Cox regression analysis was conducted in the training set to build a prognostic signature of ARG using the “glmnet” package in R. Subsequently, individualized risk scores were obtained based on the mRNA expression of selected genes, and their regression coefficients were estimated using the LASSO Cox regression analysis.

The risk score of each CRC patient was calculated with the following formula:

$$Risk\;scores=\sum_{i=1}^{n}\left(Expi\;*\;Coei\right).$$

CRC patients in the training set were divided into high- and low-risk groups according to the median risk score. The principal component analysis (PCA) was used to observe the discrimination of the patients’ samples based on the similarities in their respective groups. Kaplan–Meier analysis was used to evaluate the OS between the high- and low-risk subgroups of patients in the training set. R packages “survival,” “survminer,” and “time ROC” were used to conduct a receiver operating characteristic (ROC) curve analysis across 1-, 3-, and 5 years. Patients in the testing set were separated into low- and high-risk categories based on the median risk score from the training set. The testing set was then used to validate the anoikis-related prognostic signature.

### 2.5 Relationship between the ARG prognostic signature and clinicopathological features

The R package “survival” was used for univariate and multivariate analyses. In this analysis, variable factors included age, sex, TNM stage, and risk score. Furthermore, we used “limma” and “ggpubr” packages to analyze the relationship between risk scores and clinicopathological features. In addition, the “survival” and “survminer” packages were used for the stratified analysis of the difference in OS of patients in the high- and low-risk groups.

### 2.6 Analysis of the relationship among the ARG prognostic signature and the immune microenvironment and immunotherapy

We performed a correlation analysis of risk scores with immune cell infiltration using the CIBERSORT algorithm. Then, we downloaded the immunophenotyping file from the University of California, Santa Cruz, Xena website (https://xenabrowser.net/) and used the “limma” package to analyze the relationship between risk scores and immunophenotyping. Finally, we downloaded the CRC immune escape data from the Tumor Immune Dysfunction and Exclusion (TIDE) website (http://tide.dfci.harvard.edu/). R packages “limma,” “plyr,” “ggplot2,” and “ggpubr” were used to analyze the relationship between the risk score and immune escape in CRC.

### 2.7 Development and validation of a nomogram-based risk scoring system

Clinical characteristics and the risk score were used to build a prediction nomogram using “rms” software. Each variable was assigned a score using the nomogram scoring method, and the overall score was calculated by summing the scores for all variables in each sample.

## 3 Results

### 3.1 Identification of ARG subtypes in CRC

**FIGURE 2:**
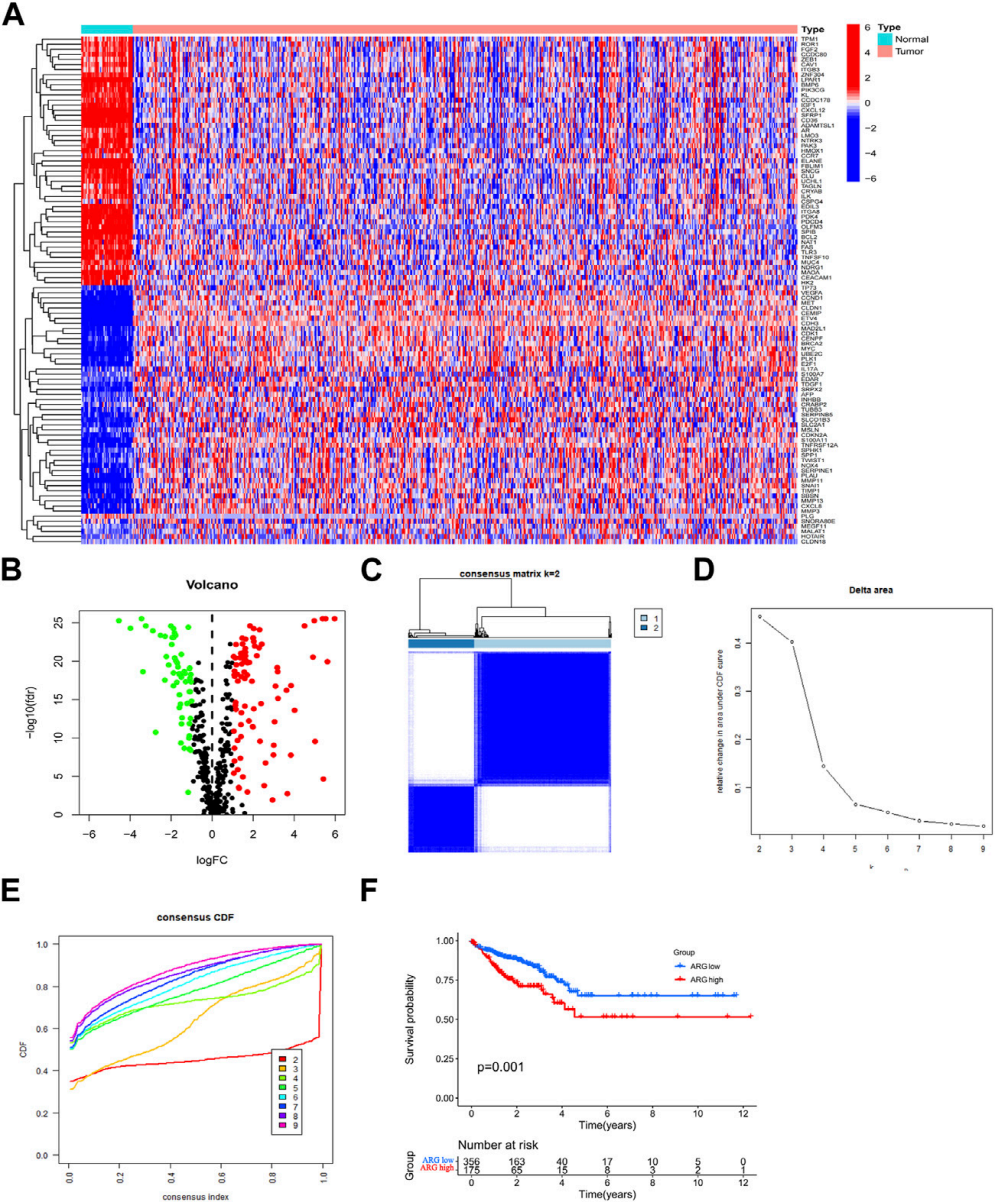
Expression of ARGs in CRC and ARG subtype identification. **(A,B)** ARGs are differentially expressed in CRC. **(C)** Two ARG subtypes in CRC were identified by consensus clustering analysis. **(D)** Relative changes in the areas under the CDF curve for k = 2–9. **(E)** Consensus clustering CDF for k = 2–9. **(F)** Survival prognostic analysis of ARG subtypes in CRC. Abbreviations: ARG, anoikis-related gene; CDF, cumulative distribution function; CRC, colorectal cancer.

To assess ARG expression patterns in CRC, clinical information from 540 CRC cohort (TCGA-COAD/READ) patients was collected from TCGA database. Moreover, 150 ARGs were found to be differentially expressed in patients with CRC (Figures 2A, B). To gain a better understanding of the expression features of ARGs in CRC, a consensus clustering approach was used to identify patients with CRC using the expression patterns of 496 ARGs. Our results showed that k = 2 was the best choice for dividing all patients into two subtypes (Figures 2C–E). Between the two subtypes, Kaplan–Meier analysis showed that different ARG subtypes of CRC exhibited significant differences in OS (Figure 2F).

### 3.2 Characteristics of different ARG subtypes

**FIGURE 3:**
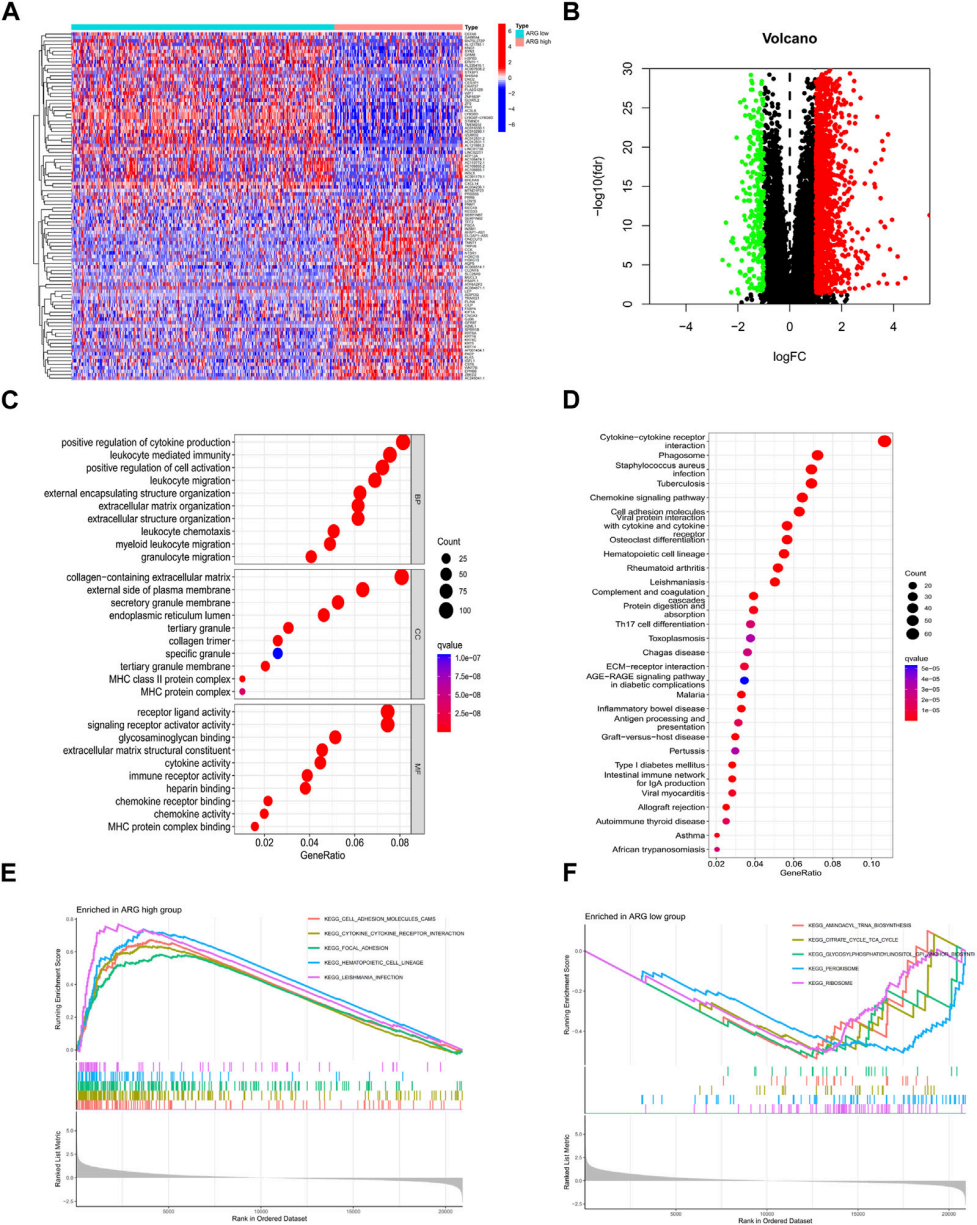
Identification and functional enrichment analysis of DEGs of ARG subtypes. **(A,B)** ARG subtypes’ DEG identification. **(C)** GO and **(D)** KEGG analysis of DEGs of ARG subtypes. **(E,F)** GSEA functional enrichment analysis of ARG subtypes. Abbreviations: ARG, anoikis-related gene; DEGs, differentially expressed genes; GO, Gene Ontology; KEGG, Kyoto Encyclopedia of Genes and Genomes; GSEA, Gene Set Enrichment Analysis.

**FIGURE 4:**
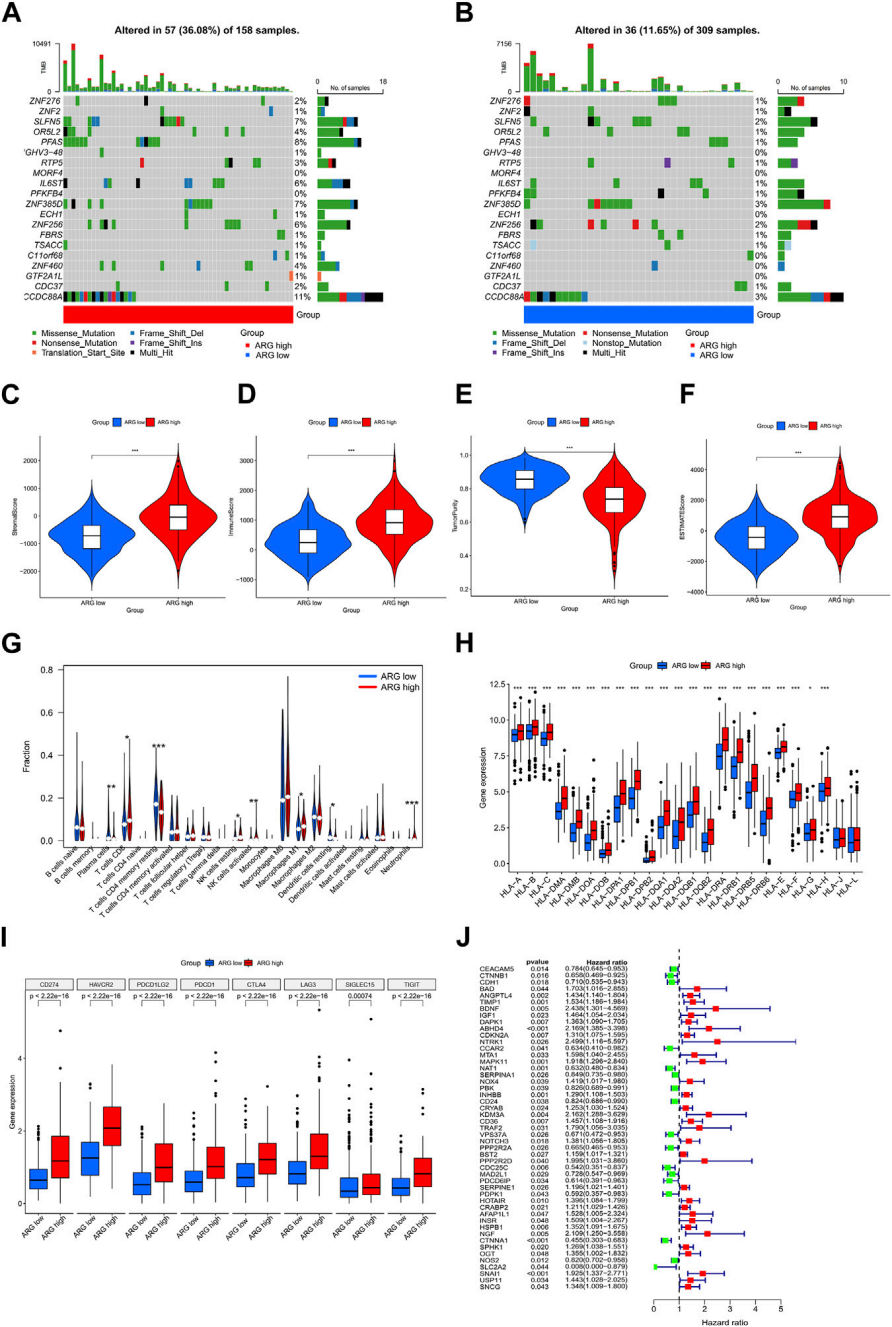
Comprehensive analysis of differences between ARG subtypes. **(A,B)** Gene mutation in ARG subtypes. Differences in stromal **(C)**, immune **(D)**, tumor purity **(E)**, and ESTIMATE **(F)** scores between ARG subtypes. **(G)** Differences in immune cell infiltration between ARG subtypes. Differential expression analysis of **(H)** HLA and **(I)** immune checkpoint-related genes between ARG subtypes. **(J)** Prognostic features of ARGs in colorectal cancer. *p < 0.05, **p < 0.01, and ***p < 0.001. Abbreviations: ARG, anoikis-related gene; ESTIMATE, Estimation of STromal and Immune cells in MAlignant Tumor tissues using Expression data; HLA, human leukocyte antigen.

Differential genes in ARG subtypes were identified for the first time (Figures 3A, B). GO and KEGG enrichment analyses showed that DEGs in ARG subtypes were significantly enriched in immune-related pathways, including positive regulation of cytokine production, leukocyte-mediated immunity, leukocyte migration, leukocyte chemotaxis, cytokine receptor, chemokine signaling pathway, antigen processing and presentation, Th17 cell differentiation, and the extracellular matrix–receptor interaction (Figures 3C, D). The DEGs in the ARG-high group were mainly concentrated in cell adhesion molecules, cytokine–receptor interaction, focal adhesion, and other signaling pathways, while in the ARG-low group, they were concentrated in amino tRNA biosynthesis non-immune signaling pathways (Figures 3E, F). We further analyzed the gene mutations between the two subtypes and compared them with the ARG-low group. The ARG-high group had a higher gene mutation frequency **(** Figures 4A, B **)**. The ESTIMATE and CIBERSORT algorithms were used to evaluate the immune microenvironment between the two ARG subtypes. ESTIMATE is a tool used for predicting tumor purity and infiltrating stromal/immune cells in tumor tissues using gene expression data (Yoshihara et al., 2013). The findings from the ESTIMATE analysis suggested that the immune (*p* < 0.001), stromal (*p* < 0.001), and ESTIMATE (*p* < 0.001) scores in the ARG-high group were markedly higher than those in the ARG-low group, while tumor purity was lower than that in the ARG-low group (Figures 4C–F). CIBERSORT is a commonly used method to calculate immune cell infiltration (Newman et al., 2019). The results showed that CD8 T cells, activated natural killer cells, M1 macrophages, and neutrophils in the ARG-high group were significantly higher than those in the ARG-low group, indicating that the ARG-high group had stronger immune activity (Figure 4G). However, some patients in the ARG-high group showed a worse prognosis. To determine the reason for the poor prognosis, we explored the expression of HLA and immune checkpoints and found that the expression of HLA antigens and immune checkpoint-related genes in the ARG-high group was higher than that in the ARG-low group (Figures 4H, I), indicating that this portion of the ARG-high group was more likely to have immune escape.

### 3.3 Construction and validation of the ARG prognostic signature

**FIGURE 5:**
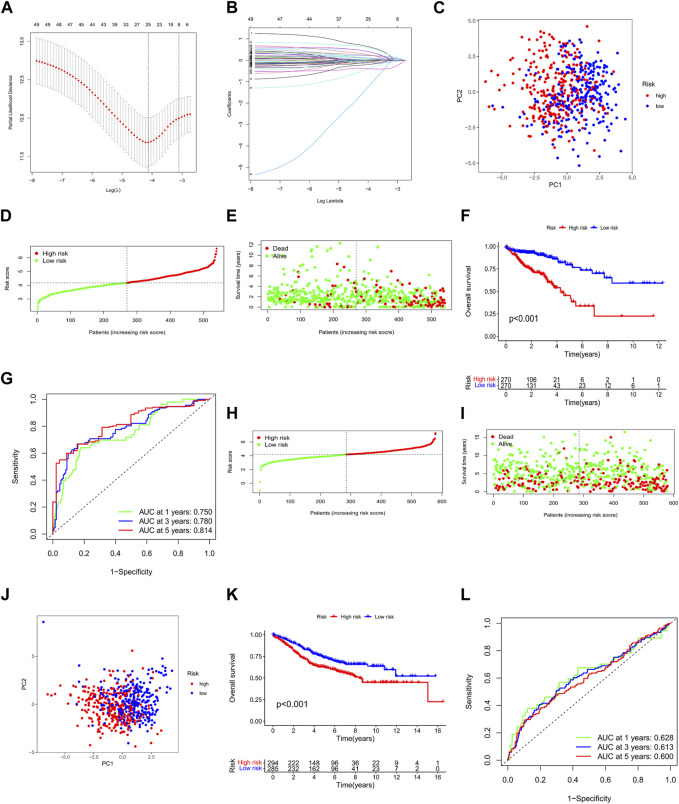
Construction and validation of the ARG prognostic signature for CRC. **(A,B)** LASSO regression analysis of prognostic ARGs. PCA analysis of CRC patients in the **(C)** training and **(J)** testing sets. Risk score analysis in the **(D)** training and **(H)** testing sets. Survival status of each patient in the **(E)** training and **(I)** testing sets. OS of patients in high- and low-risk groups in the **(F)** training and **(K)** testing cohorts. ROC curve analysis for risk scores in the **(G)** training and **(L)** testing cohorts. *p < 0.05, **p < 0.01, and ***p < 0.001. Abbreviations: CRC, colorectal cancer; PCA, principal components analysis; OS, overall survival; ROC, receiver operating characteristic; ARG, anoikis-related gene; LASSO, least absolute shrinkage and selection operator.

We used univariate Cox regression to examine the prognostic significance of 496 differentially expressed ARGs in CRC and eliminated 49 genes associated with OS (*p* < 0.05) for subsequent analysis (Figure 4J). Then, we conducted a LASSO regression analysis on 49 ARGs, resulting in the identification of 25 genes (*CTNNB1*, *BAD*, *ANGPTL4*, *TIMP1*, *BDNF*, *DAPK1*, *ABHD4*, *CCAR2*, *NAT1*, *SERPINA1*, *INHBB*, *KDM3A*, *VPS37A*, *PPP2R2A*, *PPP2R2D*, *CDC25C*, *PDPK1*, *HOTAIR*, *INSR*, *HSPB1*, *CTNNA1*, *OGT*, *NOS2*, *SLC2A2*, and *SNAI1*) (Figures 5A, B). We obtained a risk score using the following formula (−0.1308 × expression of *CTNNB1*) + (0.4366 × expression of *BAD*) + (0.1013 × expression of *ANGPTL4*) + (0.0373 × expression of *TIMP1*) + (0.6776 × expression of *BDNF*) + (0.0382 × expression of *DAPK1*) + (0.2201 × expression of *ABHD4*) + (−0.1146 × expression of *CCAR2*) + (−0.0984 × expression of *NAT1*) + (−0.021 × expression of *SERPINA1*) + (0.2488 × expression of *INHBB*) + (0.6136 × expression of *KDM3A*) + (−0.0589 × expression of *VPS37A*) + (−0.0592 × expression of *PPP2R2A*) + (0.4398 × expression of *PPP2R2D*) + (−0.2158 × expression of *CDC25C*) + (−0.4807 × expression of *PDPK1*) + (0.0346 × expression of *HOTAIR*) + (0.0721 × expression of *INSR*) + (0.1197 × expression of *HSPB1*) + (−0.3851 × expression of *CTNNA1*) + (0.2927 × expression of *OGT*) + (−0.0320 × expression of *NOS2*) + (−1.4427 × expression of *SLC2A2*) + (0.0382 × expression of *SNAI1*). Subsequently, all patients with CRC in the training set were divided into high- and low-risk groups based on the median risk score. PCA analysis revealed the discrimination of the patients’ samples based on the similarities into high- and low-risk groups (Figure 5C). In addition, we ranked the patients’ risk scores and analyzed their distribution in the training set. The survival status of CRC patients in the training set is shown as a dot plot (Figures 5D, E). The OS of patients with high-risk scores was significantly worse than that of patients in the low-risk group (*p* < 0.001) (Figure 5F). The areas under the curve of the risk signature were 0.750, 0.780, and 0.814 for the 1-, 3-, and 5-year periods, respectively (Figure 5G). Additionally, the predictive ability of the risk model was verified using a testing set. The survival score and status of CRC patients in the testing set are shown as dot plots (Figures 5H, I). Patients in the testing group were divided into two groups based on the PCA analysis (Figure 5J). The Kaplan–Meier survival curve showed that the OS of high-risk patients in the validation set was lower than that of the low-risk patients (*p* < 0.001) (Figure 5K). The areas under the curve of the testing set were 0.628, 0.613, and 0.600 for the 1-, 3-, and 5-year periods, respectively (Figure 5L).

### 3.4 Correlations between clinicopathological characteristics and the risk score

**FIGURE 6:**
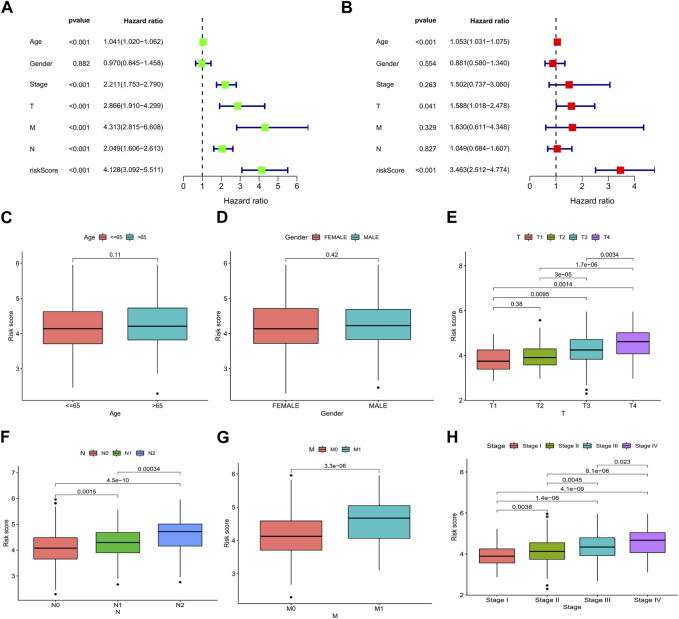
Relationship between the risk score and clinicopathological characteristics in CRC. Forest plots based on **(A)** univariate and **(B)** multivariate Cox regression analyses for OS. Correlations of risk scores and **(C)** age, **(D)** sex, **(E)** T stage, **(F)** N stage, **(G)** M stage, and **(H)** TNM stage. *p < 0.05, **p < 0.01, and ***p < 0.001. Abbreviations: ROC, receiver operating characteristic; CRC, colorectal cancer; OS, overall survival.

**FIGURE 7:**
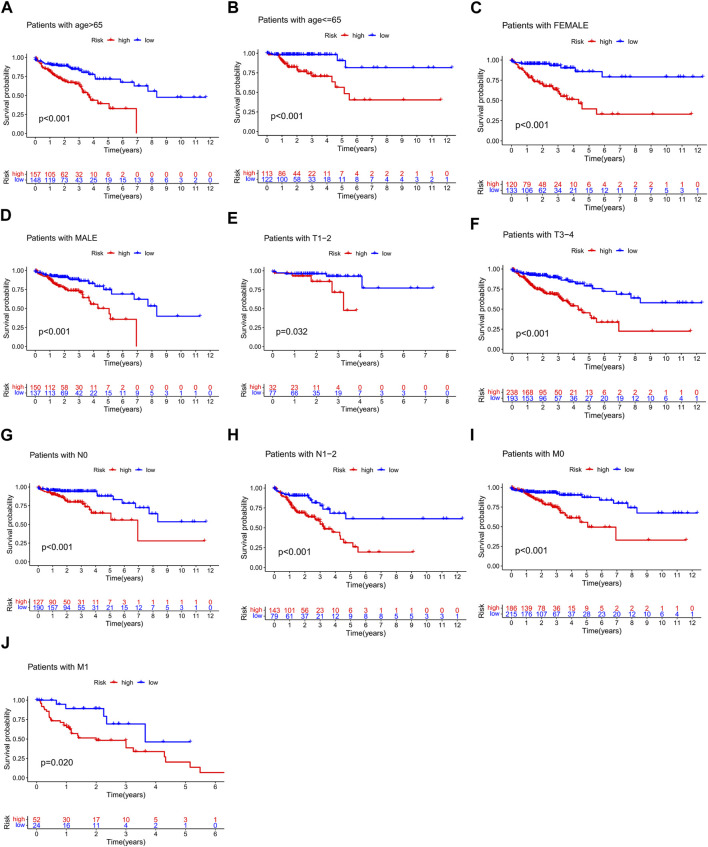
Stratified analysis of survival prognosis in high- and low-risk groups. **(A)** > 65 years, **(B)** ≤65 years, **(C)** female, **(D)** male, **(E)** T1–2, **(F)** T3–4, **(G)** N0, **(H)** N1-2, **(I)** M0, and **(J)** M1.

We analyzed the association between risk scores and clinicopathological characteristics and found that the risk score was an independent predictor of OS (Figures 6A, B; *p* < 0.001). In addition, high-risk scores were more likely to be associated with higher T-, N-, and M staging and total staging, regardless of age or sex (Figures 6C–H). The relationship between the risk score and clinicopathologic information in TCGA cohort is shown in Supplementary Table S4. In addition, the hierarchical analysis found that patients with high-risk scores tended to have poorer OS in the age, sex, and TNM stage subgroups (Figures 7A–J).

### 3.5 Relationship among the immune microenvironment, immunotherapy, and risk score

**FIGURE 8:**
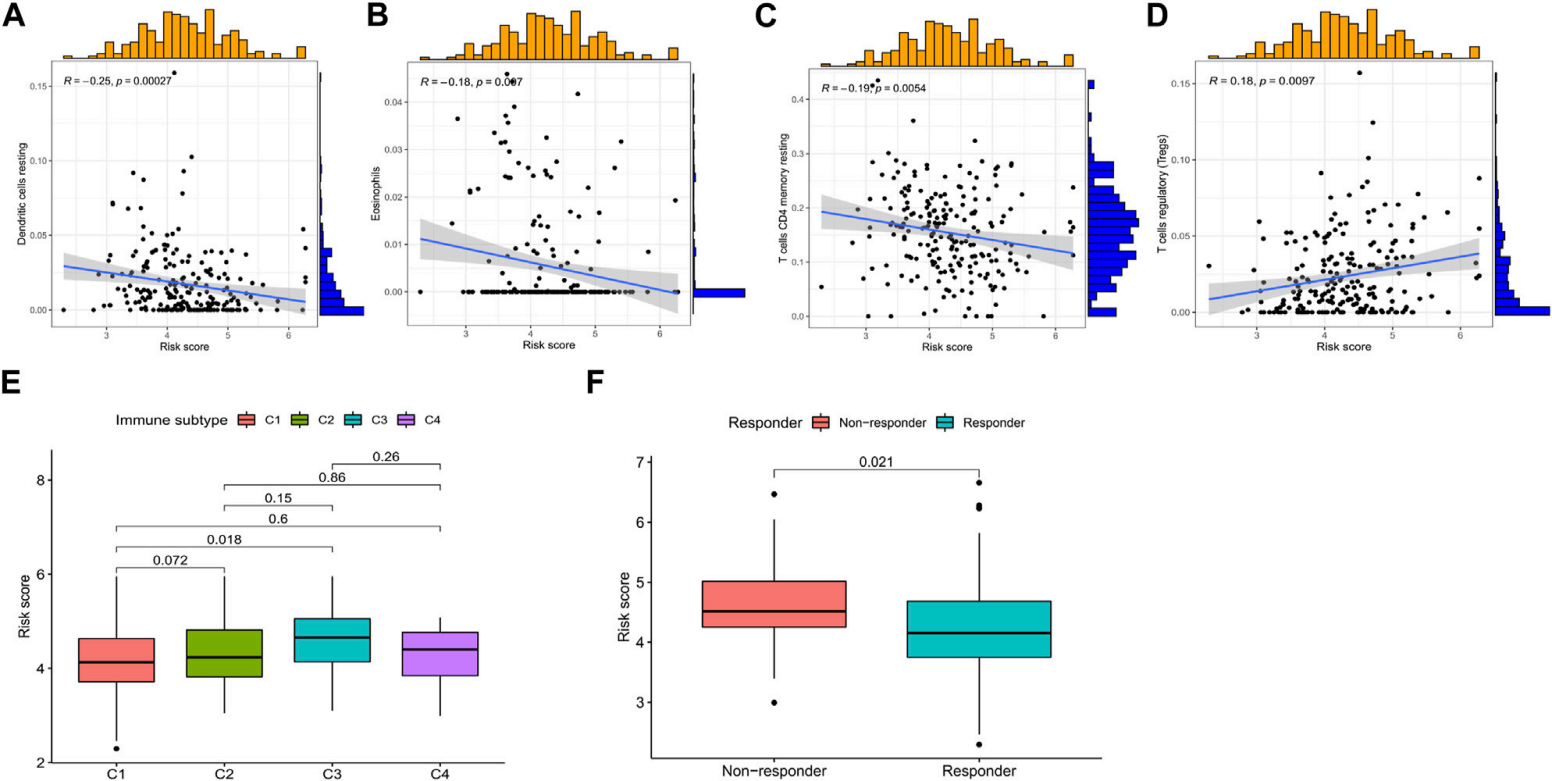
Relationship among the risk score, immune microenvironment, and immunotherapy response in CRC. Risk score associated with **(A)** dendritic cells, **(B)** eosinophils, **(C)** resting memory CD4 T cells, **(D)** regulatory T-cell infiltration, **(E)** immune subtype, and **(F)** immunotherapy response. *p < 0.05, **p < 0.01, and ***p < 0.001. Abbreviations: CRC, colorectal cancer.

Immune cell infiltration analysis revealed that the risk score was positively correlated with regulatory T cells and negatively correlated with activated dendritic cells, resting memory CD4 T cells, and eosinophils (Figures 8A–D). Risk scores were significantly different in wound healing (Immune C1) and inflammation (Immune C3) (Figure 8E). TIDE (http://tide.dfci.harvard.edu/) represents Tumor Immune Dysfunction and Exclusion (Jiang et al., 2018). In our study based on TIDE, patients with high-risk scores were more prone to non-response to immunotherapy (*p* = 0.021) (Figure 8F).

### 3.6 Development of a nomogram to predict survival

**FIGURE 9:**
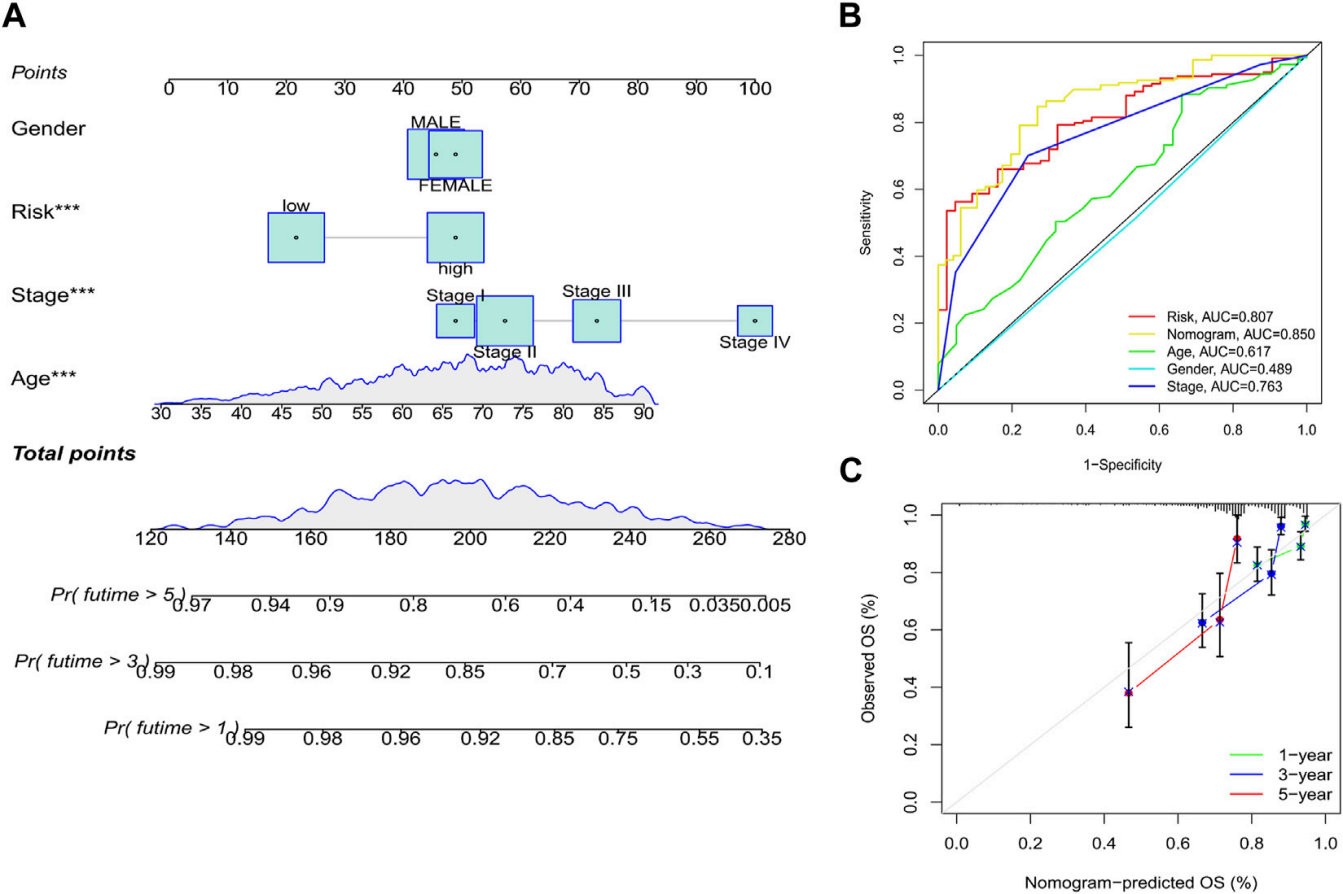
Establishment of a nomogram. **(A)** Nomogram based on the risk score combined with clinicopathological features. **(B)** ROC curve analysis of the nomogram over 5 years. **(C)** Calibration curves of the nomogram for predicting 1-, 3-, and 5-year OS. *p < 0.05, **p < 0.01, and ***p < 0.001. Abbreviations: ROC, receiver operating characteristic; OS, overall survival.

Considering that the clinical application of the risk score in predicting the OS prognosis of patients with CRC is not convenient, we established a nomogram containing the risk score and clinicopathological characteristics to predict OS (Figure 9A). Predictors included risk score, patient age, sex, and stage. In predicting the 5-year survival prognosis of patients, the nomogram ROC AUC (0.850) was better than that of the risk score and stage alone (Figure 9B). Calibration curves of the nomogram for predicting 1-, 3-, and 5-year OS in the training set suggested that the performance of the proposed nomogram was similar to the ideal model (Figure 9C).

## 4 Discussion

CRC is the third most common cause of cancer-related deaths worldwide. Among newly diagnosed CRCs, 20% are metastatic at presentation, and another 25% of localized disease later develops metastases (Biller and Schrag, 2021). Anoikis plays an essential role in various malignancies and is superior to ordinary cells in terms of autophagy (Yu et al., 2022), metabolic regulation (Wang et al., 2018; Raeisi et al., 2022), and tumor signaling regulation (Song et al., 2021; Wang et al., 2022), thus influencing a multitude of tumor-related biological processes (Taddei et al., 2012; Cao et al., 2016). Anoikis-resistant cells do not require adhesion to the extracellular matrix for survival and proliferation, and this ability is important during metastasis (Chambers et al., 2002; Nguyen et al., 2009). Consequently, anoikis resistance is a natural molecular prerequisite for the aggressive metastatic spread of cancer.

To the best of our knowledge, this is the first study to identify a link between ARGs and CRC. Genomic and clinical information related to the CRC cohort (TCGA-COAD/READ) were collected from TCGA database to further analyze the expression patterns of ARGs. Based on the expression of ARGs, we successfully identified two different molecular subtypes of colorectal cancer. DEG analysis revealed that the ARG-high group was enriched in the positive regulation of cytokine production, leukocyte-mediated immunity, regulation of cell activation, leukocyte migration, and phagosome-related signaling pathways. According to GSEA analysis, the ARG-high group was mainly enriched in cell adhesion molecules, focal adhesion, and cytokine–cytokine receptor interaction, and the ARG-low group was mainly enriched in aminoacyl tRNA biosynthesis, citrate cycle, peroxisome, ribosome, and other signaling pathways. In addition, our results suggested that the frequency of gene mutations in the ARG-high group was higher than that in the ARG-low group. These results preliminarily suggest that the differences between the ARG-high and -low groups may be related to immune-related biological processes.

Multiple components, including tumor, immune, and stromal cells, co-construct the tumor microenvironment (Hinshaw and Shevde, 2019; Xiao and Yu, 2021). Changes in different cellular components are involved in stromal remodeling (Arora and Pal, 2021), immune tolerance (Ostrand-Rosenberg, 2016), and immune escape (Simiczyjew et al., 2020), which play critical roles in tumor growth, metastasis, and treatment (Hinshaw and Shevde, 2019; Feng et al., 2022; Liu et al., 2022; Pei et al., 2022). Many studies have indicated that variations in the tumor microenvironment are a major variable in carcinogenesis (Turley et al., 2015; Seager et al., 2017; Hanahan, 2022). In the present study, substantial changes in tumor microenvironment abundance between the two clusters were detected. The ESTIMATE and CIBERSORT algorithms showed that the immune, stromal, and ESTIMATE scores were higher in the ARG-high group than those in the ARG-low group, whereas CD8^+^ cells, natural killer cells, and M1 macrophages were significantly enriched in the ARG-high group. However, CD4^+^ resting memory T cells and resting dendritic cells were enriched in the ARG-low group. CD8 T cells and natural killer cells are involved in immune homeostasis and in the regulation of autoimmune reactivity (Valipour et al., 2019; Guillerey, 2020; Lees, 2020), while M1 macrophages were previously thought to have anti-tumor effects (Boutilier and Elsawa, 2021; Gunassekaran et al., 2021). These results indicate that the immune components of the two subtypes were significantly different and that the ARG-high group exhibited superior immune activity. Activated dendritic cells have the capacity to process and perform antigen extraction through cytosolic and vacuolar pathways (Blum et al., 2013; Segura and Amigorena, 2015). Thus, the increased proportion of resting dendritic cells in the ARG-low group may have led to impaired antigen presentation. We then evaluated HLA-related genes expressed in the two clusters and found that the expression of HLA-related genes was significantly higher in the ARG-high group. We also evaluated immune checkpoint-associated gene expression in the two clusters. As expected, the expression of immune checkpoint-associated genes was significantly increased in the ARG-high group. These findings indicated that the ARG-high subtype is associated with immune activation and that these CRC patients may benefit from immune checkpoint inhibitor therapy.

The prognostic signature of CRC was established utilizing ARGs, and an optimized 25-gene model was successfully constructed. The external GEO dataset was used to verify the prognostic predictive ability of the model. We analyzed the relationship between the model’s high- and low-risk score groups and their associated clinicopathological characteristics. Multivariate Cox analysis showed that the prognostic risk signature was an independent prognostic factor, and further analysis identified that high-risk scores were associated with T, N, M, and TNM staging. A previous study performed immunogenicity analysis on more than 10,000 tumor samples from 33 cancers in TCGA and divided all tumors into six immune subtypes including wound healing, interferon-γ-dominant, inflammatory, lymphocyte-depleted, immunologically quiet, and transforming growth factor-β dominant subtypes (Huang and Fu, 2019). In our model, the C3 immune subtype had a higher risk score than the C1 immune subtype. Furthermore, risk scores were negatively correlated with dendritic cells, eosinophils, and CD4 T cells and significantly positively correlated with regulatory T cells. TIDE database-based immunotherapy scoring revealed that patients in the high-risk group were more likely to exhibit immune unresponsiveness. Furthermore, a nomogram model was constructed by combining selected clinicopathological features and risk scores, which showed good prognostic predictive power. These results suggest that the anoikis prognostic risk score signature can be used for individualized treatment of patients with CRC.

This study also had some limitations. First, this was a retrospective study with data obtained from public databases; hence, the clinicopathological characteristics were not comprehensive enough. Second, we only explored whether ARGs are related to the occurrence and development of CRC. Whether commonly used clinical treatments, such as radiotherapy and chemotherapy, can regulate the tumor immune response by inducing anoikis of tumor cells remains unclear. Thus, it is necessary to further explore the specific mechanism of action of ARGs through molecular and animal experiments.

In conclusion, we performed a comprehensive analysis based on TCGA and GEO databases, identified two anoikis subtypes, and revealed that both subtypes extensively influenced the immune microenvironment of CRC. We constructed a robust and predictive prognostic model for ARGs that are closely related to the immune microenvironment of CRC. This study supplements existing knowledge of the relationship between anoikis and immunotherapy response in CRC patients, provides a novel model for predicting the prognosis of CRC patients, and sets the foundation for the future, personalized immunotherapy in CRC patients.

## Data Availability

The datasets presented in this study can be found in online repositories. The names of the repository/repositories and accession number(s) can be found in the article/Supplementary Material.

## References

[B1] Arif A. A. Chahal D. Ladua G. K. Bhang E. Salh B. Rosenfeld G. (2021). Hereditary and inflammatory bowel disease-related early onset colorectal cancer have unique characteristics and clinical course compared with sporadic disease. Cancer Epidemiol. biomarkers Prev. 30 (10), 1785–1791. 10.1158/1055-9965.EPI-21-0507 34301727

[B2] Arora L. Pal D. (2021). Remodeling of stromal cells and immune landscape in microenvironment during tumor progression. Front. Oncol. 11, 596798. 10.3389/fonc.2021.596798 33763348 PMC7982455

[B3] Biller L. H. Schrag D. (2021). Diagnosis and treatment of metastatic colorectal cancer: A review. Jama 325 (7), 669–685. 10.1001/jama.2021.0106 33591350

[B4] Blum J. S. Wearsch P. A. Cresswell P. (2013). Pathways of antigen processing. Annu. Rev. Immunol. 31, 443–473. 10.1146/annurev-immunol-032712-095910 23298205 PMC4026165

[B5] Boutilier A. J. Elsawa S. F. (2021). Macrophage polarization States in the tumor microenvironment. Int. J. Mol. Sci. 22 (13), 6995. 10.3390/ijms22136995 34209703 PMC8268869

[B6] Boyer B. Roche S. Denoyelle M. Thiery J. P. (1997). Src and Ras are involved in separate pathways in epithelial cell scattering. Embo J. 16 (19), 5904–5913. 10.1093/emboj/16.19.5904 9312048 PMC1170221

[B7] Buchheit C. L. Weigel K. J. Schafer Z. T. (2014). Cancer cell survival during detachment from the ECM: Multiple barriers to tumour progression. Nat. Rev. Cancer 14 (9), 632–641. 10.1038/nrc3789 25098270

[B8] Cao Z. Livas T. Kyprianou N. (2016). Anoikis and EMT: Lethal "liaisons" during cancer progression. Crit. Rev. Oncog. 21 (3-4), 155–168. 10.1615/CritRevOncog.2016016955 27915969 PMC5451151

[B9] Chambers A. F. Groom A. C. MacDonald I. C. (2002). Dissemination and growth of cancer cells in metastatic sites. Nat. Rev. Cancer 2 (8), 563–572. 10.1038/nrc865 12154349

[B10] Cheng E. H. Wei M. C. Weiler S. Flavell R. A. Mak T. W. Lindsten T. (2001). BCL-2, BCL-X(L) sequester BH3 domain-only molecules preventing BAX- and BAK-mediated mitochondrial apoptosis. Mol. Cell 8 (3), 705–711. 10.1016/s1097-2765(01)00320-3 11583631

[B11] Chiarugi P. Giannoni E. (2008). Anoikis: A necessary death program for anchorage-dependent cells. Biochem. Pharmacol. 76 (11), 1352–1364. 10.1016/j.bcp.2008.07.023 18708031

[B12] Feng Y. Liu L. Li J. Huang J. Xie J. H. Menard L. (2022). Systematic characterization of the tumor microenvironment in Chinese patients with hepatocellular carcinoma highlights intratumoral B cells as a potential immunotherapy target. Oncol. Rep. 47 (2), 38. 10.3892/or.2021.8249 34958112 PMC8717124

[B13] Frisch S. M. Francis H. (1994). Disruption of epithelial cell-matrix interactions induces apoptosis. J. Cell Biol. 124 (4), 619–626. 10.1083/jcb.124.4.619 8106557 PMC2119917

[B14] Frisch S. M. Screaton R. A. (2001). Anoikis mechanisms. Curr. Opin. Cell Biol. 13 (5), 555–562. 10.1016/s0955-0674(00)00251-9 11544023

[B15] Guillerey C. (2020). NK cells in the tumor microenvironment. Adv. Exp. Med. Biol. 1273, 69–90. 10.1007/978-3-030-49270-0_4 33119876

[B16] Gunassekaran G. R. Poongkavithai Vadevoo S. M. Baek M. C. Lee B. (2021). M1 macrophage exosomes engineered to foster M1 polarization and target the IL-4 receptor inhibit tumor growth by reprogramming tumor-associated macrophages into M1-like macrophages. Biomaterials 278, 121137. 10.1016/j.biomaterials.2021.121137 34560422

[B17] Hanahan D. (2022). Hallmarks of cancer: New dimensions. Cancer Discov. 12 (1), 31–46. 10.1158/2159-8290.CD-21-1059 35022204

[B18] Hinshaw D. C. Shevde L. A. (2019). The tumor microenvironment innately modulates cancer progression. Cancer Res. 79 (18), 4557–4566. 10.1158/0008-5472.CAN-18-3962 31350295 PMC6744958

[B19] Huang T. X. Fu L. (2019). The immune landscape of esophageal cancer. Cancer Commun. Lond. Engl. 39 (1), 79. 10.1186/s40880-019-0427-z PMC6878621 31771653

[B20] Jiang P. Gu S. Pan D. Fu J. Sahu A. Hu X. (2018). Signatures of T cell dysfunction and exclusion predict cancer immunotherapy response. Nat. Med. 24 (10), 1550–1558. 10.1038/s41591-018-0136-1 30127393 PMC6487502

[B21] Khwaja A. Rodriguez-Viciana P. Wennström S. Warne P. H. Downward J. (1997). Matrix adhesion and Ras transformation both activate a phosphoinositide 3-OH kinase and protein kinase B/Akt cellular survival pathway. Embo J. 16 (10), 2783–2793. 10.1093/emboj/16.10.2783 9184223 PMC1169887

[B22] Lees J. R. (2020). CD8+ T cells: The past and future of immune regulation. Cell. Immunol. 357, 104212. 10.1016/j.cellimm.2020.104212 32979764

[B23] Liu X. Hoft D. F. Peng G. (2022). Tumor microenvironment metabolites directing T cell differentiation and function. Trends Immunol. 43 (2), 132–147. 10.1016/j.it.2021.12.004 34973923 PMC8810659

[B24] Morozevich G. E. Kozlova N. I. Chubukina A. N. Berman A. E. (2003). Role of integrin alphavbeta3 in substrate-dependent apoptosis of human intestinal carcinoma cells. Biochem. (Mosc) 68 (4), 416–423. 10.1023/a:1023699829927 12765524

[B25] Newman A. M. Steen C. B. Liu C. L. Gentles A. J. Chaudhuri A. A. Scherer F. (2019). Determining cell type abundance and expression from bulk tissues with digital cytometry. Nat. Biotechnol. 37 (7), 773–782. 10.1038/s41587-019-0114-2 31061481 PMC6610714

[B26] Nguyen D. X. Bos P. D. Massagué J. (2009). Metastasis: From dissemination to organ-specific colonization. Nat. Rev. Cancer 9 (4), 274–284. 10.1038/nrc2622 19308067

[B27] Ostrand-Rosenberg S. (2016). Tolerance and immune suppression in the tumor microenvironment. Cell. Immunol. 299, 23–29. 10.1016/j.cellimm.2015.09.011 26435343 PMC4698223

[B28] Paoli P. Giannoni E. Chiarugi P. (2013). Anoikis molecular pathways and its role in cancer progression. Biochim. Biophys. Acta 1833 (12), 3481–3498. 10.1016/j.bbamcr.2013.06.026 23830918

[B29] Pei P. Shen W. Zhang Y. Zhang Y. Qi Z. Zhou H. (2022). Radioactive nano-oxygen generator enhance anti-tumor radio-immunotherapy by regulating tumor microenvironment and reducing proliferation. Biomaterials 280, 121326. 10.1016/j.biomaterials.2021.121326 34953386

[B30] Raeisi M. Zehtabi M. Velaei K. Fayyazpour P. Aghaei N. Mehdizadeh A. (2022). Anoikis in cancer: The role of lipid signaling. Cell Biol. Int. 46 (11), 1717–1728. 10.1002/cbin.11896 36030535

[B31] Seager R. J. Hajal C. Spill F. Kamm R. D. Zaman M. H. (2017). Dynamic interplay between tumour, stroma and immune system can drive or prevent tumour progression. Convergent Sci. Phys. Oncol. 3, 034002. 10.1088/2057-1739/aa7e86 PMC6070160 30079253

[B32] Segura E. Amigorena S. (2015). Cross-presentation in mouse and human dendritic cells. Adv. Immunol. 127, 1–31. 10.1016/bs.ai.2015.03.002 26073982

[B33] Simiczyjew A. Dratkiewicz E. Mazurkiewicz J. Ziętek M. Matkowski R. Nowak D. (2020). The influence of tumor microenvironment on immune escape of melanoma. Int. J. Mol. Sci. 21 (21), 8359. 10.3390/ijms21218359 33171792 PMC7664679

[B34] Song J. Liu Y. Liu F. Zhang L. Li G. Yuan C. (2021). The 14-3-3σ protein promotes HCC anoikis resistance by inhibiting EGFR degradation and thereby activating the EGFR-dependent ERK1/2 signaling pathway. Theranostics 11 (3), 996–1015. 10.7150/thno.51646 33391517 PMC7738881

[B35] Sousa B. Pereira J. Marques R. Grilo L. F. Pereira S. P. Sardão V. A. (2020). P-cadherin induces anoikis-resistance of matrix-detached breast cancer cells by promoting pentose phosphate pathway and decreasing oxidative stress. Biochim. Biophys. Acta Mol. Basis Dis. 1866 (12), 165964. 10.1016/j.bbadis.2020.165964 32920119

[B36] Sung H. Ferlay J. Siegel R. L. Laversanne M. Soerjomataram I. Jemal A. (2021). Global cancer statistics 2020: GLOBOCAN estimates of incidence and mortality worldwide for 36 cancers in 185 countries. CA a cancer J. Clin. 71 (3), 209–249. 10.3322/caac.21660 33538338

[B37] Taddei M. L. Giannoni E. Fiaschi T. Chiarugi P. (2012). Anoikis: An emerging hallmark in health and diseases. J. pathology 226 (2), 380–393. 10.1002/path.3000 21953325

[B38] Turley S. J. Cremasco V. Astarita J. L. (2015). Immunological hallmarks of stromal cells in the tumour microenvironment. Nat. Rev. Immunol. 15 (11), 669–682. 10.1038/nri3902 26471778

[B39] Valipour B. Velaei K. Abedelahi A. Karimipour M. Darabi M. Charoudeh H. N. (2019). NK cells: An attractive candidate for cancer therapy. J. Cell. physiology 234 (11), 19352–19365. 10.1002/jcp.28657 30993712

[B40] Wang L. N. Zhang Z. T. Wang L. Wei H. X. Zhang T. Zhang L. M. (2022). TGF-β1/SH2B3 axis regulates anoikis resistance and EMT of lung cancer cells by modulating JAK2/STAT3 and SHP2/Grb2 signaling pathways. Cell death Dis. 13 (5), 472. 10.1038/s41419-022-04890-x 35589677 PMC9120066

[B41] Wang Y. N. Zeng Z. L. Lu J. Wang Y. Liu Z. X. He M. M. (2018). CPT1A-mediated fatty acid oxidation promotes colorectal cancer cell metastasis by inhibiting anoikis. Oncogene 37 (46), 6025–6040. 10.1038/s41388-018-0384-z 29995871

[B42] Xiao Y. Yu D. (2021). Tumor microenvironment as a therapeutic target in cancer. Pharmacol. Ther. 221, 107753. 10.1016/j.pharmthera.2020.107753 33259885 PMC8084948

[B43] Yamamoto T. Kawada K. Obama K. (2021). Inflammation-related biomarkers for the prediction of prognosis in colorectal cancer patients. Int. J. Mol. Sci. 22 (15), 8002. 10.3390/ijms22158002 34360768 PMC8348168

[B44] Yoshihara K. Shahmoradgoli M. Martínez E. Vegesna R. Kim H. Torres-Garcia W. (2013). Inferring tumour purity and stromal and immune cell admixture from expression data. Nat. Commun. 4, 2612. 10.1038/ncomms3612 24113773 PMC3826632

[B45] Yu Y. Song Y. Cheng L. Chen L. Liu B. Lu D. (2022). CircCEMIP promotes anoikis-resistance by enhancing protective autophagy in prostate cancer cells. J. Exp. Clin. cancer Res. CR 41 (1), 188. 10.1186/s13046-022-02381-7 35655258 PMC9161511

