# Supplementary material for: A novel anoikis-related gene prognostic signature and its correlation with the immune microenvironment in colorectal cancer
Source: Front Genet. 2023 May 30;14:1186862. doi: 10.3389/fgene.2023.1186862 (PMC10265740; doi:10.3389/fgene.2023.1186862)
Supplement: Supplementary file 1 [file DataSheet1.docx]

Supplementary Material

A Novel Anoikis-related Gene Prognostic Signature and its Correlation with Immune Microenvironment in Colorectal Cancer

**Yu Xiao^1†^, Han Zhou^1†^, Yiran Chen^1^, Libin Liu^1^ Qian Wu^1^, Hui Li^1^, Peicheng Lin^1^, Jinluan Li^1^, Junxin Wu^1*^, Lirui Tang^1*^**

**^1^ Department of Radiation Oncology, Clinical Oncology School of Fujian Medical University, Fujian Cancer Hospital, Fuzhou, China**

**^†^ These authors contributed equally to this work.**

**^*^ Correspondence:**

**Lirui Tang
E-mail: tlr330306938@pku.edu.cn**

**Junxin Wu
E-mail:** [**junxinwu@126.com**](mailto:junxinwu@126.com)

# Supplementary Figures and Tables

For more information on Supplementary Material and for details on the different file types accepted, please see [here](https://www.frontiersin.org/guidelines/author-guidelines#supplementary-material).

**Supplementary Table 1.** Clinicopathological characteristics of colorectal cancer patients in the TCGA database.

| Variables |  | Number |
| --- | --- | --- |
| Total |  | 540 |
| Status | alive | 431 |
|  | death | 109 |
| Age | <=65 | 236 |
|  | >65 | 304 |
| Gender | female | 253 |
|  | male | 287 |
| Stage | stageI | 93 |
|  | stageII | 207 |
|  | stageIII | 148 |
|  | stageIV | 77 |
|  | unknow | 15 |
| T | T1 | 16 |
|  | T2 | 93 |
|  | T3 | 368 |
|  | T4 | 63 |
| N | N0 | 317 |
|  | N1 | 129 |
|  | N2 | 93 |
|  | unknow | 1 |
| M | M0 | 401 |
|  | M1 | 76 |
|  | unknow | 63 |

**Supplementary Table 2.** Clinicopathological characteristics of colorectal cancer patient in the GEO database.

| Variables |  | Number |
| --- | --- | --- |
| Total |  | 585 |
| Gender | Female | 263 |
|  | male | 322 |
| Stage | 0 | 4 |
|  | I | 38 |
|  | II | 271 |
|  | III | 210 |
|  | IV | 60 |
|  | unknow | 2 |
| T | Tis | 3 |
|  | 0 | 1 |
|  | 1 | 12 |
|  | 2 | 49 |
|  | 3 | 379 |
|  | 4 | 119 |
|  | unknow | 22 |
| N | N0 | 314 |
|  | N1 | 137 |
|  | N2 | 100 |
|  | N3 | 6 |
|  | N+ | 6 |
|  | unknow | 22 |
| M | M0 | 499 |
|  | M1 | 61 |
|  | unknow | 25 |
| Status | alive | 385 |
|  | death | 194 |
|  | unknow | 6 |

**Supplementary Table 3.** Anoikis-related genes with relevence scores greater than 0.4 in GeneCards website.

| Gene Symbol | Description | Relevance score |
| --- | --- | --- |
| BRMS1 | BRMS1 Transcriptional Repressor And Anoikis Regulator | 14.7101965 |
| PTK2 | Protein Tyrosine Kinase 2 | 7.287514687 |
| NTRK2 | Neurotrophic Receptor Tyrosine Kinase 2 | 7.26254034 |
| BCL2L11 | BCL2 Like 11 | 6.691599369 |
| SRC | SRC Proto-Oncogene, Non-Receptor Tyrosine Kinase | 6.184272289 |
| CEACAM6 | CEA Cell Adhesion Molecule 6 | 6.103184223 |
| CAV1 | Caveolin 1 | 5.463297844 |
| AKT1 | AKT Serine/Threonine Kinase 1 | 5.407460213 |
| ITGB1 | Integrin Subunit Beta 1 | 5.002956867 |
| CEACAM5 | CEA Cell Adhesion Molecule 5 | 4.655790806 |
| EGFR | Epidermal Growth Factor Receptor | 4.615612507 |
| BCL2 | BCL2 Apoptosis Regulator | 4.541404724 |
| CASP8 | Caspase 8 | 4.488767147 |
| SIK1 | Salt Inducible Kinase 1 | 4.375074387 |
| PTRH2 | Peptidyl-TRNA Hydrolase 2 | 4.192895889 |
| STAT3 | Signal Transducer And Activator Of Transcription 3 | 4.140684605 |
| TLE1 | TLE Family Member 1, Transcriptional Corepressor | 4.070988178 |
| DAPK2 | Death Associated Protein Kinase 2 | 3.9884758 |
| CTNNB1 | Catenin Beta 1 | 3.976930857 |
| ZNF304 | Zinc Finger Protein 304 | 3.940119267 |
| MAPK1 | Mitogen-Activated Protein Kinase 1 | 3.734493017 |
| BMF | Bcl2 Modifying Factor | 3.732539892 |
| ITGA5 | Integrin Subunit Alpha 5 | 3.677546978 |
| TP53 | Tumor Protein P53 | 3.639735699 |
| MCL1 | MCL1 Apoptosis Regulator, BCL2 Family Member | 3.579083204 |
| BCL2L1 | BCL2 Like 1 | 3.362038851 |
| CASP3 | Caspase 3 | 3.119940281 |
| CDH1 | Cadherin 1 | 3.059575081 |
| BAD | BCL2 Associated Agonist Of Cell Death | 2.963050365 |
| PIK3CA | Phosphatidylinositol-4,5-Bisphosphate 3-Kinase Catalytic Subunit Alpha | 2.942048311 |
| PAK1 | P21 (RAC1) Activated Kinase 1 | 2.930860281 |
| ITGAV | Integrin Subunit Alpha V | 2.875283718 |
| FN1 | Fibronectin 1 | 2.821427584 |
| MAPK3 | Mitogen-Activated Protein Kinase 3 | 2.729858875 |
| PTGS2 | Prostaglandin-Endoperoxide Synthase 2 | 2.692126036 |
| BAX | BCL2 Associated X, Apoptosis Regulator | 2.548128128 |
| BCAR1 | BCAR1 Scaffold Protein, Cas Family Member | 2.548128128 |
| PTEN | Phosphatase And Tensin Homolog | 2.518462658 |
| ERBB2 | Erb-B2 Receptor Tyrosine Kinase 2 | 2.433978081 |
| ANGPTL4 | Angiopoietin Like 4 | 2.417241573 |
| PDK4 | Pyruvate Dehydrogenase Kinase 4 | 2.415361166 |
| CYCS | Cytochrome C, Somatic | 2.339439392 |
| BRAF | B-Raf Proto-Oncogene, Serine/Threonine Kinase | 2.335298777 |
| YAP1 | Yes1 Associated Transcriptional Regulator | 2.332169533 |
| ANKRD13C | Ankyrin Repeat Domain 13C | 2.329536915 |
| ITGA2 | Integrin Subunit Alpha 2 | 2.298167467 |
| ANXA5 | Annexin A5 | 2.265911579 |
| BIRC5 | Baculoviral IAP Repeat Containing 5 | 2.255683661 |
| MTOR | Mechanistic Target Of Rapamycin Kinase | 2.249702692 |
| TIMP1 | TIMP Metallopeptidase Inhibitor 1 | 2.245340347 |
| BDNF | Brain Derived Neurotrophic Factor | 2.22146225 |
| CSPG4 | Chondroitin Sulfate Proteoglycan 4 | 2.195114136 |
| BSG | Basigin (Ok Blood Group) | 2.195114136 |
| AKT2 | AKT Serine/Threonine Kinase 2 | 2.18259716 |
| STK11 | Serine/Threonine Kinase 11 | 2.150352716 |
| IGF1 | Insulin Like Growth Factor 1 | 2.147692442 |
| IGF1R | Insulin Like Growth Factor 1 Receptor | 2.146956682 |
| ITGA6 | Integrin Subunit Alpha 6 | 2.109769821 |
| ILK | Integrin Linked Kinase | 2.083154917 |
| CFLAR | CASP8 And FADD Like Apoptosis Regulator | 2.083021879 |
| RHOA | Ras Homolog Family Member A | 2.068578482 |
| HIF1A | Hypoxia Inducible Factor 1 Subunit Alpha | 2.064971447 |
| DAP3 | Death Associated Protein 3 | 2.055300236 |
| MYBBP1A | MYB Binding Protein 1a | 2.020877361 |
| TLE5 | TLE Family Member 5, Transcriptional Modulator | 1.998172402 |
| ITGA3 | Integrin Subunit Alpha 3 | 1.998085022 |
| PTK2B | Protein Tyrosine Kinase 2 Beta | 1.99505043 |
| CCND1 | Cyclin D1 | 1.981372356 |
| CTTN | Cortactin | 1.981372356 |
| CALR | Calreticulin | 1.944714546 |
| ATF4 | Activating Transcription Factor 4 | 1.944714546 |
| CDCP1 | CUB Domain Containing Protein 1 | 1.931936264 |
| PLAUR | Plasminogen Activator, Urokinase Receptor | 1.907844424 |
| SKP2 | S-Phase Kinase Associated Protein 2 | 1.907844424 |
| CHEK2 | Checkpoint Kinase 2 | 1.907212973 |
| HGF | Hepatocyte Growth Factor | 1.883365631 |
| E2F1 | E2F Transcription Factor 1 | 1.87997818 |
| EGF | Epidermal Growth Factor | 1.870716572 |
| PIK3CG | Phosphatidylinositol-4,5-Bisphosphate 3-Kinase Catalytic Subunit Gamma | 1.867439747 |
| ITGB4 | Integrin Subunit Beta 4 | 1.855637074 |
| DAPK1 | Death Associated Protein Kinase 1 | 1.845545173 |
| MAPK8 | Mitogen-Activated Protein Kinase 8 | 1.839446306 |
| PIK3R1 | Phosphoinositide-3-Kinase Regulatory Subunit 1 | 1.820626736 |
| PIK3R3 | Phosphoinositide-3-Kinase Regulatory Subunit 3 | 1.815660119 |
| MAP2K1 | Mitogen-Activated Protein Kinase Kinase 1 | 1.796159506 |
| CXCL12 | C-X-C Motif Chemokine Ligand 12 | 1.77687037 |
| LGALS3 | Galectin 3 | 1.741451263 |
| FBXW7-AS1 | FBXW7 Antisense RNA 1 | 1.733860254 |
| BAK1 | BCL2 Antagonist/Killer 1 | 1.730095983 |
| ABHD4 | Abhydrolase Domain Containing 4, N-Acyl Phospholipase B | 1.706411362 |
| CD44 | CD44 Molecule (Indian Blood Group) | 1.702935219 |
| ITGA4 | Integrin Subunit Alpha 4 | 1.689652681 |
| FADD | Fas Associated Via Death Domain | 1.689652681 |
| PHLDA2 | Pleckstrin Homology Like Domain Family A Member 2 | 1.689652681 |
| TGFB1 | Transforming Growth Factor Beta 1 | 1.68550384 |
| HMCN1 | Hemicentin 1 | 1.68550384 |
| MMP2 | Matrix Metallopeptidase 2 | 1.672284126 |
| CEBPB | CCAAT Enhancer Binding Protein Beta | 1.672284126 |
| CEMIP | Cell Migration Inducing Hyaluronidase 1 | 1.672284126 |
| CDKN3 | Cyclin Dependent Kinase Inhibitor 3 | 1.666557789 |
| CBL | Cbl Proto-Oncogene | 1.654233575 |
| CASP9 | Caspase 9 | 1.654233575 |
| SFN | Stratifin | 1.654233575 |
| MTDH | Metadherin | 1.654233575 |
| PRKCA | Protein Kinase C Alpha | 1.635413885 |
| TNFRSF10B | TNF Receptor Superfamily Member 10b | 1.635413885 |
| CXCL8 | C-X-C Motif Chemokine Ligand 8 | 1.635413885 |
| MIR200C | MicroRNA 200c | 1.635413885 |
| AR | Androgen Receptor | 1.618417978 |
| CDKN2A | Cyclin Dependent Kinase Inhibitor 2A | 1.615717649 |
| CPT1A | Carnitine Palmitoyltransferase 1A | 1.615717649 |
| PIK3CB | Phosphatidylinositol-4,5-Bisphosphate 3-Kinase Catalytic Subunit Beta | 1.615717649 |
| CLDN1 | Claudin 1 | 1.615717649 |
| MIR204 | MicroRNA 204 | 1.615717649 |
| MIR26A1 | MicroRNA 26a-1 | 1.615717649 |
| CDKN1A | Cyclin Dependent Kinase Inhibitor 1A | 1.595009208 |
| CDKN1B | Cyclin Dependent Kinase Inhibitor 1B | 1.595009208 |
| KLF12 | KLF Transcription Factor 12 | 1.595009208 |
| NTRK1 | Neurotrophic Receptor Tyrosine Kinase 1 | 1.573605061 |
| PLAU | Plasminogen Activator, Urokinase | 1.573114634 |
| MYC | MYC Proto-Oncogene, BHLH Transcription Factor | 1.573114634 |
| SMAD4 | SMAD Family Member 4 | 1.573114634 |
| PLK1 | Polo Like Kinase 1 | 1.573114634 |
| MUC1 | Mucin 1, Cell Surface Associated | 1.573114634 |
| LGALS1 | Galectin 1 | 1.573114634 |
| PYCARD | PYD And CARD Domain Containing | 1.573114634 |
| SESN2 | Sestrin 2 | 1.573114634 |
| ITGB3 | Integrin Subunit Beta 3 | 1.568463564 |
| KRAS | KRAS Proto-Oncogene, GTPase | 1.568463564 |
| THBS1 | Thrombospondin 1 | 1.549803257 |
| BID | BH3 Interacting Domain Death Agonist | 1.549803257 |
| HRAS | HRas Proto-Oncogene, GTPase | 1.535089016 |
| CDK11B | Cyclin Dependent Kinase 11B | 1.52475822 |
| CDK11A | Cyclin Dependent Kinase 11A | 1.52475822 |
| XIAP | X-Linked Inhibitor Of Apoptosis | 1.51812768 |
| PPARG | Peroxisome Proliferator Activated Receptor Gamma | 1.497523308 |
| IL6 | Interleukin 6 | 1.497523308 |
| MIR145 | MicroRNA 145 | 1.497523308 |
| CCR7 | C-C Motif Chemokine Receptor 7 | 1.467398405 |
| MSLN | Mesothelin | 1.467398405 |
| RAC1 | Rac Family Small GTPase 1 | 1.461561203 |
| GRHL2 | Grainyhead Like Transcription Factor 2 | 1.461561203 |
| BIRC3 | Baculoviral IAP Repeat Containing 3 | 1.448278546 |
| NOTCH1 | Notch Receptor 1 | 1.438148737 |
| RHOG | Ras Homolog Family Member G | 1.436138988 |
| CCAR2 | Cell Cycle And Apoptosis Regulator 2 | 1.436138988 |
| NQO1 | NAD(P)H Quinone Dehydrogenase 1 | 1.433205247 |
| MMP13 | Matrix Metallopeptidase 13 | 1.398545146 |
| FAS | Fas Cell Surface Death Receptor | 1.395646811 |
| MTA1 | Metastasis Associated 1 | 1.395646811 |
| MYO5A | Myosin VA | 1.392645836 |
| EDA2R | Ectodysplasin A2 Receptor | 1.392645836 |
| CCN6 | Cellular Communication Network Factor 6 | 1.392645836 |
| MMP9 | Matrix Metallopeptidase 9 | 1.374343395 |
| ABL1 | ABL Proto-Oncogene 1, Non-Receptor Tyrosine Kinase | 1.374343395 |
| MAPK11 | Mitogen-Activated Protein Kinase 11 | 1.374343395 |
| SOD2 | Superoxide Dismutase 2 | 1.374343395 |
| PTHLH | Parathyroid Hormone Like Hormone | 1.370601773 |
| PDGFB | Platelet Derived Growth Factor Subunit B | 1.353635073 |
| GLI2 | GLI Family Zinc Finger 2 | 1.353635073 |
| EZH2 | Enhancer Of Zeste 2 Polycomb Repressive Complex 2 Subunit | 1.352576733 |
| RIPK1 | Receptor Interacting Serine/Threonine Kinase 1 | 1.351372838 |
| CXCR4 | C-X-C Motif Chemokine Receptor 4 | 1.343366861 |
| HMGA1 | High Mobility Group AT-Hook 1 | 1.331740618 |
| SIK2 | Salt Inducible Kinase 2 | 1.331740618 |
| TNFSF10 | TNF Superfamily Member 10 | 1.331740618 |
| ANGPTL2 | Angiopoietin Like 2 | 1.313241959 |
| S100A4 | S100 Calcium Binding Protein A4 | 1.308429122 |
| NTF3 | Neurotrophin 3 | 1.308429122 |
| ETV4 | ETS Variant Transcription Factor 4 | 1.308429122 |
| MIR21 | MicroRNA 21 | 1.308429122 |
| MIR124-1 | MicroRNA 124-1 | 1.308429122 |
| HTRA1 | HtrA Serine Peptidase 1 | 1.283384085 |
| LATS1 | Large Tumor Suppressor Kinase 1 | 1.283384085 |
| CEACAM3 | CEA Cell Adhesion Molecule 3 | 1.283384085 |
| EIF2AK3 | Eukaryotic Translation Initiation Factor 2 Alpha Kinase 3 | 1.280375481 |
| LAMC2 | Laminin Subunit Gamma 2 | 1.280375481 |
| LAMA3 | Laminin Subunit Alpha 3 | 1.280375481 |
| LAMB3 | Laminin Subunit Beta 3 | 1.280375481 |
| CDH2 | Cadherin 2 | 1.260679126 |
| CSNK2A1 | Casein Kinase 2 Alpha 1 | 1.260679126 |
| EDIL3 | EGF Like Repeats And Discoidin Domains 3 | 1.260679126 |
| ZEB2 | Zinc Finger E-Box Binding Homeobox 2 | 1.256149173 |
| TLN1 | Talin 1 | 1.256149173 |
| EPHA2 | EPH Receptor A2 | 1.239970803 |
| SIRT3 | Sirtuin 3 | 1.239970803 |
| OLFM3 | Olfactomedin 3 | 1.239970803 |
| CLU | Clusterin | 1.226024389 |
| SPINK1 | Serine Peptidase Inhibitor Kazal Type 1 | 1.226024389 |
| CPEB2 | Cytoplasmic Polyadenylation Element Binding Protein 2 | 1.226024389 |
| NAT1 | N-Acetyltransferase 1 | 1.218076229 |
| TSG101 | Tumor Susceptibility 101 | 1.218076229 |
| MIR200A | MicroRNA 200a | 1.218076229 |
| MIR6744 | MicroRNA 6744 | 1.218076229 |
| SERPINA1 | Serpin Family A Member 1 | 1.212176919 |
| AKT3 | AKT Serine/Threonine Kinase 3 | 1.194764853 |
| RELA | RELA Proto-Oncogene, NF-KB Subunit | 1.194764853 |
| TNFRSF1A | TNF Receptor Superfamily Member 1A | 1.194764853 |
| FASLG | Fas Ligand | 1.194764853 |
| AFP | Alpha Fetoprotein | 1.194764853 |
| ITGA8 | Integrin Subunit Alpha 8 | 1.194764853 |
| NOX4 | NADPH Oxidase 4 | 1.194764853 |
| PBK | PDZ Binding Kinase | 1.194764853 |
| SATB1 | SATB Homeobox 1 | 1.194764853 |
| CD63 | CD63 Molecule | 1.194764853 |
| EEF1A1 | Eukaryotic Translation Elongation Factor 1 Alpha 1 | 1.194764853 |
| LTB4R2 | Leukotriene B4 Receptor 2 | 1.194764853 |
| MAVS | Mitochondrial Antiviral Signaling Protein | 1.194764853 |
| HRC | Histidine Rich Calcium Binding Protein | 1.194764853 |
| CCN2 | Cellular Communication Network Factor 2 | 1.191831112 |
| RHOB | Ras Homolog Family Member B | 1.191831112 |
| PPP1R13B | Protein Phosphatase 1 Regulatory Subunit 13B | 1.191831112 |
| PLG | Plasminogen | 1.185631275 |
| MET | MET Proto-Oncogene, Receptor Tyrosine Kinase | 1.184291959 |
| RAF1 | Raf-1 Proto-Oncogene, Serine/Threonine Kinase | 1.169719696 |
| PARP1 | Poly(ADP-Ribose) Polymerase 1 | 1.169719696 |
| PRKCQ | Protein Kinase C Theta | 1.169719696 |
| BRCA2 | BRCA2 DNA Repair Associated | 1.169719696 |
| RB1 | RB Transcriptional Corepressor 1 | 1.169719696 |
| SP1 | Sp1 Transcription Factor | 1.169719696 |
| HAVCR2 | Hepatitis A Virus Cellular Receptor 2 | 1.169719696 |
| DOCK1 | Dedicator Of Cytokinesis 1 | 1.169719696 |
| VTN | Vitronectin | 1.169719696 |
| INHBB | Inhibin Subunit Beta B | 1.169719696 |
| PDCD4 | Programmed Cell Death 4 | 1.169719696 |
| PRPF4B | Pre-MRNA Processing Factor 4B | 1.169719696 |
| RANBP9 | RAN Binding Protein 9 | 1.169719696 |
| SESN1 | Sestrin 1 | 1.169719696 |
| SESN3 | Sestrin 3 | 1.169719696 |
| CD24 | CD24 Molecule | 1.169719696 |
| ZBTB7A | Zinc Finger And BTB Domain Containing 7A | 1.169719696 |
| MIR141 | MicroRNA 141 | 1.169719696 |
| ELANE | Elastase, Neutrophil Expressed | 1.151271701 |
| KDR | Kinase Insert Domain Receptor | 1.142484903 |
| MDM2 | MDM2 Proto-Oncogene | 1.142484903 |
| NFE2L2 | NFE2 Like BZIP Transcription Factor 2 | 1.142484903 |
| ZEB1 | Zinc Finger E-Box Binding Homeobox 1 | 1.142484903 |
| KL | Klotho | 1.142484903 |
| PRKCI | Protein Kinase C Iota | 1.142484903 |
| CRYAB | Crystallin Alpha B | 1.142484903 |
| EPHB6 | EPH Receptor B6 | 1.142484903 |
| FGF2 | Fibroblast Growth Factor 2 | 1.142484903 |
| HK2 | Hexokinase 2 | 1.142484903 |
| LTF | Lactotransferrin | 1.142484903 |
| IQGAP1 | IQ Motif Containing GTPase Activating Protein 1 | 1.142484903 |
| MGAT5 | Alpha-1,6-Mannosylglycoprotein 6-Beta-N-Acetylglucosaminyltransferase | 1.142484903 |
| SDCBP | Syndecan Binding Protein | 1.142484903 |
| ABHD2 | Abhydrolase Domain Containing 2, Acylglycerol Lipase | 1.142484903 |
| SPIB | Spi-B Transcription Factor | 1.142484903 |
| TRIM31 | Tripartite Motif Containing 31 | 1.142484903 |
| MIR1827 | MicroRNA 1827 | 1.142484903 |
| PDGFRB | Platelet Derived Growth Factor Receptor Beta | 1.112360001 |
| PLAT | Plasminogen Activator, Tissue Type | 1.112360001 |
| TLR3 | Toll Like Receptor 3 | 1.112360001 |
| NRAS | NRAS Proto-Oncogene, GTPase | 1.112360001 |
| ROCK1 | Rho Associated Coiled-Coil Containing Protein Kinase 1 | 1.112360001 |
| PAK4 | P21 (RAC1) Activated Kinase 4 | 1.112360001 |
| VEGFA | Vascular Endothelial Growth Factor A | 1.112360001 |
| CASP10 | Caspase 10 | 1.112360001 |
| PIN1 | Peptidylprolyl Cis/Trans Isomerase, NIMA-Interacting 1 | 1.112360001 |
| IL1RAP | Interleukin 1 Receptor Accessory Protein | 1.112360001 |
| UBE2C | Ubiquitin Conjugating Enzyme E2 C | 1.112360001 |
| YWHAZ | Tyrosine 3-Monooxygenase/Tryptophan 5-Monooxygenase Activation Protein Zeta | 1.112360001 |
| TWIST1 | Twist Family BHLH Transcription Factor 1 | 1.112360001 |
| BMP6 | Bone Morphogenetic Protein 6 | 1.112360001 |
| BNIP3L | BCL2 Interacting Protein 3 Like | 1.112360001 |
| ELK1 | ETS Transcription Factor ELK1 | 1.112360001 |
| KDM3A | Lysine Demethylase 3A | 1.112360001 |
| PRDX4 | Peroxiredoxin 4 | 1.112360001 |
| BNIP3 | BCL2 Interacting Protein 3 | 1.112360001 |
| LMO3 | LIM Domain Only 3 | 1.112360001 |
| ZNF32 | Zinc Finger Protein 32 | 1.112360001 |
| MIR200B | MicroRNA 200b | 1.112360001 |
| MIR525 | MicroRNA 525 | 1.112360001 |
| MIR363 | MicroRNA 363 | 1.112360001 |
| TUBB3 | Tubulin Beta 3 Class III | 1.098413587 |
| HSP90B1 | Heat Shock Protein 90 Beta Family Member 1 | 1.098413587 |
| SLC2A1 | Solute Carrier Family 2 Member 1 | 1.078166842 |
| HMOX1 | Heme Oxygenase 1 | 1.078166842 |
| PTPN11 | Protein Tyrosine Phosphatase Non-Receptor Type 11 | 1.078166842 |
| PRKACA | Protein Kinase CAMP-Activated Catalytic Subunit Alpha | 1.078166842 |
| PAK3 | P21 (RAC1) Activated Kinase 3 | 1.078166842 |
| CD36 | CD36 Molecule | 1.078166842 |
| PIK3R2 | Phosphoinositide-3-Kinase Regulatory Subunit 2 | 1.078166842 |
| PPP2CA | Protein Phosphatase 2 Catalytic Subunit Alpha | 1.078166842 |
| CASP6 | Caspase 6 | 1.078166842 |
| CDH3 | Cadherin 3 | 1.078166842 |
| EEF2K | Eukaryotic Elongation Factor 2 Kinase | 1.078166842 |
| LRP1 | LDL Receptor Related Protein 1 | 1.078166842 |
| PAK2 | P21 (RAC1) Activated Kinase 2 | 1.078166842 |
| PTK6 | Protein Tyrosine Kinase 6 | 1.078166842 |
| LPAR1 | Lysophosphatidic Acid Receptor 1 | 1.078166842 |
| CEACAM1 | CEA Cell Adhesion Molecule 1 | 1.078166842 |
| GDF2 | Growth Differentiation Factor 2 | 1.078166842 |
| GLO1 | Glyoxalase I | 1.078166842 |
| IL17A | Interleukin 17A | 1.078166842 |
| RBL2 | RB Transcriptional Corepressor Like 2 | 1.078166842 |
| SIRPA | Signal Regulatory Protein Alpha | 1.078166842 |
| TRAF2 | TNF Receptor Associated Factor 2 | 1.078166842 |
| ADCY10 | Adenylate Cyclase 10 | 1.078166842 |
| VPS37A | VPS37A Subunit Of ESCRT-I | 1.078166842 |
| TNFRSF12A | TNF Receptor Superfamily Member 12A | 1.078166842 |
| APOBEC3G | Apolipoprotein B MRNA Editing Enzyme Catalytic Subunit 3G | 1.078166842 |
| BAG1 | BAG Cochaperone 1 | 1.078166842 |
| COL13A1 | Collagen Type XIII Alpha 1 Chain | 1.078166842 |
| MNX1 | Motor Neuron And Pancreas Homeobox 1 | 1.078166842 |
| RAD9A | RAD9 Checkpoint Clamp Component A | 1.078166842 |
| IFI27 | Interferon Alpha Inducible Protein 27 | 1.078166842 |
| MEGF11 | Multiple EGF Like Domains 11 | 1.078166842 |
| ITPRIP | Inositol 1,4,5-Trisphosphate Receptor Interacting Protein | 1.078166842 |
| BCL2L15 | BCL2 Like 15 | 1.078166842 |
| SNAI2 | Snail Family Transcriptional Repressor 2 | 1.050074577 |
| PTPN1 | Protein Tyrosine Phosphatase Non-Receptor Type 1 | 1.037607431 |
| NOTCH3 | Notch Receptor 3 | 1.037607431 |
| GLUD1 | Glutamate Dehydrogenase 1 | 1.037607431 |
| SIRT1 | Sirtuin 1 | 1.037607431 |
| FASN | Fatty Acid Synthase | 1.037607431 |
| MYH9 | Myosin Heavy Chain 9 | 1.037607431 |
| RPS6KB1 | Ribosomal Protein S6 Kinase B1 | 1.037607431 |
| TPM1 | Tropomyosin 1 | 1.037607431 |
| PPP2R1A | Protein Phosphatase 2 Scaffold Subunit Aalpha | 1.037607431 |
| COL4A2 | Collagen Type IV Alpha 2 Chain | 1.037607431 |
| CTNND1 | Catenin Delta 1 | 1.037607431 |
| CD151 | CD151 Molecule (Raph Blood Group) | 1.037607431 |
| MMP11 | Matrix Metallopeptidase 11 | 1.037607431 |
| ARHGEF7 | Rho Guanine Nucleotide Exchange Factor 7 | 1.037607431 |
| PPP2R2A | Protein Phosphatase 2 Regulatory Subunit Balpha | 1.037607431 |
| SEMA7A | Semaphorin 7A (John Milton Hagen Blood Group) | 1.037607431 |
| PPP2R5A | Protein Phosphatase 2 Regulatory Subunit B'Alpha | 1.037607431 |
| BST2 | Bone Marrow Stromal Cell Antigen 2 | 1.037607431 |
| CCN1 | Cellular Communication Network Factor 1 | 1.037607431 |
| PPP2R2D | Protein Phosphatase 2 Regulatory Subunit Bdelta | 1.037607431 |
| CCDC178 | Coiled-Coil Domain Containing 178 | 1.037607431 |
| MIR10A | MicroRNA 10a | 1.037607431 |
| MIR30B | MicroRNA 30b | 1.037607431 |
| MIR30C1 | MicroRNA 30c-1 | 1.037607431 |
| SHC1 | SHC Adaptor Protein 1 | 1.016698599 |
| BUB1 | BUB1 Mitotic Checkpoint Serine/Threonine Kinase | 0.984749258 |
| CDC25C | Cell Division Cycle 25C | 0.984749258 |
| CDK1 | Cyclin Dependent Kinase 1 | 0.984749258 |
| ITGB5 | Integrin Subunit Beta 5 | 0.984749258 |
| SETD2 | SET Domain Containing 2, Histone Lysine Methyltransferase | 0.984749258 |
| BUB3 | BUB3 Mitotic Checkpoint Protein | 0.984749258 |
| FER | FER Tyrosine Kinase | 0.984749258 |
| TP73 | Tumor Protein P73 | 0.984749258 |
| SLCO1B3 | Solute Carrier Organic Anion Transporter Family Member 1B3 | 0.984749258 |
| TDGF1 | Teratocarcinoma-Derived Growth Factor 1 | 0.984749258 |
| DLG1 | Discs Large MAGUK Scaffold Protein 1 | 0.984749258 |
| EDAR | Ectodysplasin A Receptor | 0.984749258 |
| MAD2L1 | Mitotic Arrest Deficient 2 Like 1 | 0.984749258 |
| BCL2L2 | BCL2 Like 2 | 0.984749258 |
| PDCD6IP | Programmed Cell Death 6 Interacting Protein | 0.984749258 |
| SH3GLB1 | SH3 Domain Containing GRB2 Like, Endophilin B1 | 0.984749258 |
| SCRIB | Scribble Planar Cell Polarity Protein | 0.984749258 |
| DYNLL2 | Dynein Light Chain LC8-Type 2 | 0.984749258 |
| TSC2 | TSC Complex Subunit 2 | 0.958521605 |
| BAG4 | BAG Cochaperone 4 | 0.920354962 |
| MAP3K7 | Mitogen-Activated Protein Kinase Kinase Kinase 7 | 0.917962193 |
| F10 | Coagulation Factor X | 0.857138515 |
| F3 | Coagulation Factor III, Tissue Factor | 0.857138515 |
| ADAMTSL1 | ADAMTS Like 1 | 0.857138515 |
| SERPINB1 | Serpin Family B Member 1 | 0.857138515 |
| MIR181A1 | MicroRNA 181a-1 | 0.857138515 |
| MAP3K1 | Mitogen-Activated Protein Kinase Kinase Kinase 1 | 0.83795011 |
| CTBP1 | C-Terminal Binding Protein 1 | 0.83795011 |
| CEACAM4 | CEA Cell Adhesion Molecule 4 | 0.803756893 |
| PXN | Paxillin | 0.79161489 |
| MALAT1 | Metastasis Associated Lung Adenocarcinoma Transcript 1 | 0.787873149 |
| IKBKG | Inhibitor Of Nuclear Factor Kappa B Kinase Regulatory Subunit Gamma | 0.737493277 |
| TFDP1 | Transcription Factor Dp-1 | 0.737493277 |
| CRYBA1 | Crystallin Beta A1 | 0.737493277 |
| SERPINE1 | Serpin Family E Member 1 | 0.730513453 |
| FOXO3 | Forkhead Box O3 | 0.729288697 |
| ACTG1 | Actin Gamma 1 | 0.710339308 |
| ARHGDIA | Rho GDP Dissociation Inhibitor Alpha | 0.710339308 |
| EZR | Ezrin | 0.710339308 |
| SLC39A6 | Solute Carrier Family 39 Member 6 | 0.710339308 |
| BIN1 | Bridging Integrator 1 | 0.70065546 |
| TIAM1 | TIAM Rac1 Associated GEF 1 | 0.70065546 |
| PDPK1 | 3-Phosphoinositide Dependent Protein Kinase 1 | 0.696320236 |
| SMAD7 | SMAD Family Member 7 | 0.673420548 |
| NTRK3 | Neurotrophic Receptor Tyrosine Kinase 3 | 0.643295765 |
| RHOC | Ras Homolog Family Member C | 0.643295765 |
| CASP2 | Caspase 2 | 0.635347724 |
| TNC | Tenascin C | 0.612036288 |
| IRF6 | Interferon Regulatory Factor 6 | 0.612036288 |
| HOTAIR | HOX Transcript Antisense RNA | 0.609102547 |
| GNE | Glucosamine (UDP-N-Acetyl)-2-Epimerase/N-Acetylmannosamine Kinase | 0.586991191 |
| XAF1 | XIAP Associated Factor 1 | 0.586991191 |
| SFRP1 | Secreted Frizzled Related Protein 1 | 0.582728565 |
| MAP2K2 | Mitogen-Activated Protein Kinase Kinase 2 | 0.568543077 |
| CSK | C-Terminal Src Kinase | 0.568543077 |
| PIK3C2B | Phosphatidylinositol-4-Phosphate 3-Kinase Catalytic Subunit Type 2 Beta | 0.568543077 |
| TAGLN | Transgelin | 0.568543077 |
| ENDOG | Endonuclease G | 0.568543077 |
| FOXC2 | Forkhead Box C2 | 0.568543077 |
| RACK1 | Receptor For Activated C Kinase 1 | 0.568543077 |
| ARHGDIB | Rho GDP Dissociation Inhibitor Beta | 0.568543077 |
| FBLIM1 | Filamin Binding LIM Protein 1 | 0.568543077 |
| CCDC80 | Coiled-Coil Domain Containing 80 | 0.568543077 |
| PRKD1 | Protein Kinase D1 | 0.559756279 |
| LDHA | Lactate Dehydrogenase A | 0.529631495 |
| ANXA2 | Annexin A2 | 0.529631495 |
| SPP1 | Secreted Phosphoprotein 1 | 0.529631495 |
| SMARCE1 | SWI/SNF Related, Matrix Associated, Actin Dependent Regulator Of Chromatin, Subfamily E, Member 1 | 0.529631495 |
| QSOX1 | Quiescin Sulfhydryl Oxidase 1 | 0.529631495 |
| RBFOX2 | RNA Binding Fox-1 Homolog 2 | 0.529631495 |
| RPS6KA3 | Ribosomal Protein S6 Kinase A3 | 0.495438248 |
| CDC42 | Cell Division Cycle 42 | 0.495438248 |
| MAOA | Monoamine Oxidase A | 0.495438248 |
| PIP5K1C | Phosphatidylinositol-4-Phosphate 5-Kinase Type 1 Gamma | 0.495438248 |
| ATF2 | Activating Transcription Factor 2 | 0.495438248 |
| JUP | Junction Plakoglobin | 0.495438248 |
| NDRG1 | N-Myc Downstream Regulated 1 | 0.495438248 |
| NKX2-1 | NK2 Homeobox 1 | 0.495438248 |
| OCLN | Occludin | 0.495438248 |
| CRABP2 | Cellular Retinoic Acid Binding Protein 2 | 0.495438248 |
| ID2 | Inhibitor Of DNA Binding 2 | 0.495438248 |
| CEACAM8 | CEA Cell Adhesion Molecule 8 | 0.495438248 |
| PITPNC1 | Phosphatidylinositol Transfer Protein Cytoplasmic 1 | 0.495438248 |
| AFAP1L1 | Actin Filament Associated Protein 1 Like 1 | 0.495438248 |
| INSR | Insulin Receptor | 0.454878807 |
| HSPB1 | Heat Shock Protein Family B (Small) Member 1 | 0.454878807 |
| NGF | Nerve Growth Factor | 0.454878807 |
| PCNA | Proliferating Cell Nuclear Antigen | 0.454878807 |
| GSK3B | Glycogen Synthase Kinase 3 Beta | 0.454878807 |
| TP63 | Tumor Protein P63 | 0.454878807 |
| KRT14 | Keratin 14 | 0.454878807 |
| SPHK1 | Sphingosine Kinase 1 | 0.454878807 |
| CTNNA1 | Catenin Alpha 1 | 0.454878807 |
| EHMT2 | Euchromatic Histone Lysine Methyltransferase 2 | 0.454878807 |
| OGT | O-Linked N-Acetylglucosamine (GlcNAc) Transferase | 0.454878807 |
| RAC3 | Rac Family Small GTPase 3 | 0.454878807 |
| SIRT6 | Sirtuin 6 | 0.454878807 |
| ACP1 | Acid Phosphatase 1 | 0.454878807 |
| FOXA1 | Forkhead Box A1 | 0.454878807 |
| STK38 | Serine/Threonine Kinase 38 | 0.454878807 |
| RHOQ | Ras Homolog Family Member Q | 0.454878807 |
| ONECUT1 | One Cut Homeobox 1 | 0.454878807 |
| S100A7 | S100 Calcium Binding Protein A7 | 0.454878807 |
| SRSF3 | Serine And Arginine Rich Splicing Factor 3 | 0.454878807 |
| MUC4 | Mucin 4, Cell Surface Associated | 0.454878807 |
| GKN1 | Gastrokine 1 | 0.454878807 |
| MIR107 | MicroRNA 107 | 0.454878807 |
| MIR630 | MicroRNA 630 | 0.454878807 |
| DNMT1 | DNA Methyltransferase 1 | 0.402020693 |
| LCK | LCK Proto-Oncogene, Src Family Tyrosine Kinase | 0.402020693 |
| MERTK | MER Proto-Oncogene, Tyrosine Kinase | 0.402020693 |
| UCHL1 | Ubiquitin C-Terminal Hydrolase L1 | 0.402020693 |
| CDK2 | Cyclin Dependent Kinase 2 | 0.402020693 |
| MMP3 | Matrix Metallopeptidase 3 | 0.402020693 |
| ACTB | Actin Beta | 0.402020693 |
| BRCA1 | BRCA1 DNA Repair Associated | 0.402020693 |
| SLC2A2 | Solute Carrier Family 2 Member 2 | 0.402020693 |
| NOS2 | Nitric Oxide Synthase 2 | 0.402020693 |
| USP9X | Ubiquitin Specific Peptidase 9 X-Linked | 0.402020693 |
| ROR1 | Receptor Tyrosine Kinase Like Orphan Receptor 1 | 0.402020693 |
| FYN | FYN Proto-Oncogene, Src Family Tyrosine Kinase | 0.402020693 |
| HSPA1A | Heat Shock Protein Family A (Hsp70) Member 1A | 0.402020693 |
| HTRA2 | HtrA Serine Peptidase 2 | 0.402020693 |
| SNAI1 | Snail Family Transcriptional Repressor 1 | 0.402020693 |
| C5AR1 | Complement C5a Receptor 1 | 0.402020693 |
| LATS2 | Large Tumor Suppressor Kinase 2 | 0.402020693 |
| PRDM1 | PR/SET Domain 1 | 0.402020693 |
| SKI | SKI Proto-Oncogene | 0.402020693 |
| TPP2 | Tripeptidyl Peptidase 2 | 0.402020693 |
| XRCC5 | X-Ray Repair Cross Complementing 5 | 0.402020693 |
| CLDN18 | Claudin 18 | 0.402020693 |
| SPTA1 | Spectrin Alpha, Erythrocytic 1 | 0.402020693 |
| THY1 | Thy-1 Cell Surface Antigen | 0.402020693 |
| TJP1 | Tight Junction Protein 1 | 0.402020693 |
| CDX2 | Caudal Type Homeobox 2 | 0.402020693 |
| CENPF | Centromere Protein F | 0.402020693 |
| DOK2 | Docking Protein 2 | 0.402020693 |
| S100A11 | S100 Calcium Binding Protein A11 | 0.402020693 |
| SERPINB5 | Serpin Family B Member 5 | 0.402020693 |
| IKZF3 | IKAROS Family Zinc Finger 3 | 0.402020693 |
| SNCG | Synuclein Gamma | 0.402020693 |
| USP11 | Ubiquitin Specific Peptidase 11 | 0.402020693 |
| HOXA10 | Homeobox A10 | 0.402020693 |
| LGALS8 | Galectin 8 | 0.402020693 |
| SRPX2 | Sushi Repeat Containing Protein X-Linked 2 | 0.402020693 |
| SLPI | Secretory Leukocyte Peptidase Inhibitor | 0.402020693 |
| HTRA3 | HtrA Serine Peptidase 3 | 0.402020693 |
| EFHD2 | EF-Hand Domain Family Member D2 | 0.402020693 |
| IRX1 | Iroquois Homeobox 1 | 0.402020693 |
| CXCL14 | C-X-C Motif Chemokine Ligand 14 | 0.402020693 |
| KIF18A | Kinesin Family Member 18A | 0.402020693 |
| ZG16B | Zymogen Granule Protein 16B | 0.402020693 |
| SBSN | Suprabasin | 0.402020693 |
| MIR223 | MicroRNA 223 | 0.402020693 |
| MIR503 | MicroRNA 503 | 0.402020693 |
| MIR99A | MicroRNA 99a | 0.402020693 |
| MIR451A | MicroRNA 451a | 0.402020693 |
| MIR7-1 | MicroRNA 7-1 | 0.402020693 |
| SNORA80E | Small Nucleolar RNA, H/ACA Box 80E | 0.402020693 |

**Supplementary Table 4.** The relationship between risk score and clinicopathologic information in TCGA cohort.

| Id | Age | Gender | Stage | T | M | N | Risk group |
| --- | --- | --- | --- | --- | --- | --- | --- |
| TCGA-3L-AA1B | 61 | FEMALE | Stage I | T2 | M0 | N0 | low |
| TCGA-4N-A93T | 67 | MALE | Stage III | T4 | M0 | N1 | high |
| TCGA-4T-AA8H | 42 | FEMALE | Stage II | T3 | unknow | N0 | low |
| TCGA-5M-AAT4 | 74 | MALE | Stage IV | T3 | M1 | N0 | high |
| TCGA-5M-AAT6 | 40 | FEMALE | Stage IV | T4 | M1 | N2 | high |
| TCGA-5M-AATE | 76 | MALE | Stage II | T3 | M0 | N0 | high |
| TCGA-A6-2671 | 85 | MALE | Stage IV | T3 | M1 | N2 | low |
| TCGA-A6-2672 | 82 | FEMALE | Stage III | T3 | M0 | N1 | high |
| TCGA-A6-2674 | 71 | MALE | Stage IV | T3 | M1 | N2 | high |
| TCGA-A6-2675 | 78 | MALE | Stage II | T3 | unknow | N0 | low |
| TCGA-A6-2676 | 75 | FEMALE | Stage II | T4 | M0 | N0 | high |
| TCGA-A6-2677 | 68 | FEMALE | Stage III | T3 | M0 | N2 | high |
| TCGA-A6-2678 | 43 | FEMALE | Stage III | T3 | M0 | N1 | low |
| TCGA-A6-2679 | 73 | FEMALE | Stage II | T3 | unknow | N0 | high |
| TCGA-A6-2680 | 72 | FEMALE | Stage II | T3 | M0 | N0 | low |
| TCGA-A6-2681 | 73 | FEMALE | Stage II | T3 | M0 | N0 | low |
| TCGA-A6-2682 | 70 | MALE | Stage IV | T4 | M1 | N1 | high |
| TCGA-A6-2683 | 57 | FEMALE | Stage IV | T4 | M1 | N0 | high |
| TCGA-A6-2684 | 75 | FEMALE | Stage I | T2 | M0 | N0 | high |
| TCGA-A6-2685 | 48 | FEMALE | Stage II | T3 | M0 | N0 | high |
| TCGA-A6-2686 | 81 | FEMALE | Stage II | T3 | M0 | N0 | high |
| TCGA-A6-3807 | 53 | FEMALE | Stage III | T3 | M0 | N2 | high |
| TCGA-A6-3808 | 73 | MALE | Stage II | T3 | M0 | N0 | high |
| TCGA-A6-3809 | 71 | FEMALE | Stage II | T4 | M0 | N0 | low |
| TCGA-A6-3810 | 62 | MALE | Stage II | T3 | M0 | N0 | low |
| TCGA-A6-4105 | 79 | MALE | Stage II | T3 | M0 | N0 | high |
| TCGA-A6-4107 | 57 | FEMALE | Stage III | T3 | M0 | N1 | low |
| TCGA-A6-5656 | 74 | MALE | Stage I | T2 | M0 | N0 | low |
| TCGA-A6-5657 | 65 | MALE | Stage III | T3 | M0 | N1 | high |
| TCGA-A6-5659 | 82 | MALE | Stage I | T2 | M0 | N0 | low |
| TCGA-A6-5660 | 73 | MALE | Stage III | T3 | M0 | N2 | high |
| TCGA-A6-5661 | 80 | FEMALE | Stage II | T3 | M0 | N0 | low |
| TCGA-A6-5662 | 46 | MALE | Stage IV | T3 | M1 | N2 | low |
| TCGA-A6-5664 | 80 | MALE | Stage III | T4 | unknow | N2 | low |
| TCGA-A6-5665 | 84 | FEMALE | Stage II | T3 | M0 | N0 | high |
| TCGA-A6-5666 | 78 | MALE | Stage II | T4 | M0 | N0 | high |
| TCGA-A6-5667 | 40 | FEMALE | Stage III | T3 | unknow | N1 | low |
| TCGA-A6-6137 | 55 | MALE | Stage III | T3 | M0 | N1 | high |
| TCGA-A6-6138 | 61 | MALE | Stage I | T2 | M0 | N0 | low |
| TCGA-A6-6140 | 62 | MALE | Stage II | T3 | M0 | N0 | low |
| TCGA-A6-6141 | 31 | MALE | Stage II | T3 | M0 | N0 | low |
| TCGA-A6-6142 | 56 | FEMALE | Stage IV | T3 | M1 | N1 | high |
| TCGA-A6-6648 | 56 | MALE | Stage IV | T3 | M1 | N0 | low |
| TCGA-A6-6649 | 66 | MALE | Stage III | T3 | M0 | N1 | high |
| TCGA-A6-6650 | 69 | FEMALE | Stage II | T3 | M0 | N0 | low |
| TCGA-A6-6651 | 55 | FEMALE | Stage III | T3 | unknow | N1 | high |
| TCGA-A6-6652 | 59 | MALE | Stage IV | T3 | M1 | N0 | high |
| TCGA-A6-6653 | 82 | MALE | Stage I | T2 | M0 | N0 | high |
| TCGA-A6-6654 | 65 | FEMALE | Stage III | T3 | M0 | N1 | high |
| TCGA-A6-6780 | 74 | MALE | Stage II | T3 | unknow | N0 | low |
| TCGA-A6-6781 | 43 | MALE | Stage III | T4 | unknow | N1 | high |
| TCGA-A6-6782 | 82 | MALE | Stage II | T4 | unknow | N0 | high |
| TCGA-A6-A565 | 34 | FEMALE | Stage III | T3 | unknow | N2 | high |
| TCGA-A6-A566 | 55 | FEMALE | Stage III | T4 | M0 | N1 | high |
| TCGA-A6-A567 | 56 | MALE | Stage IV | T3 | M1 | N1 | high |
| TCGA-A6-A56B | 57 | MALE | Stage III | T3 | M0 | N1 | low |
| TCGA-A6-A5ZU | 59 | MALE | Stage III | T3 | M0 | N1 | high |
| TCGA-AA-3489 | 75 | MALE | Stage II | T3 | M0 | N0 | high |
| TCGA-AA-3492 | 90 | FEMALE | Stage II | T3 | M0 | N0 | low |
| TCGA-AA-3494 | 55 | MALE | Stage IV | T3 | M1 | N0 | low |
| TCGA-AA-3495 | 79 | MALE | Stage I | T2 | M0 | N0 | low |
| TCGA-AA-3496 | 83 | FEMALE | Stage II | T3 | M0 | N0 | high |
| TCGA-AA-3502 | 73 | MALE | Stage I | T2 | M0 | N0 | low |
| TCGA-AA-3506 | 77 | MALE | Stage I | T2 | M0 | N0 | low |
| TCGA-AA-3509 | 54 | FEMALE | Stage II | T3 | M0 | N0 | low |
| TCGA-AA-3510 | 70 | MALE | Stage II | T3 | M0 | N0 | high |
| TCGA-AA-3511 | 64 | MALE | Stage II | T4 | M0 | N0 | low |
| TCGA-AA-3514 | 81 | FEMALE | Stage I | T2 | M0 | N0 | low |
| TCGA-AA-3516 | 74 | FEMALE | Stage III | T3 | M0 | N2 | high |
| TCGA-AA-3517 | 60 | MALE | Stage II | T3 | M0 | N0 | high |
| TCGA-AA-3518 | 81 | FEMALE | Stage II | T3 | M0 | N0 | low |
| TCGA-AA-3519 | 63 | MALE | Stage III | T3 | M0 | N1 | low |
| TCGA-AA-3520 | 86 | FEMALE | Stage II | T3 | M0 | N0 | low |
| TCGA-AA-3522 | 67 | MALE | Stage II | T3 | M0 | N0 | low |
| TCGA-AA-3524 | 85 | MALE | Stage II | T3 | M0 | N0 | low |
| TCGA-AA-3525 | 90 | MALE | Stage III | T3 | M0 | N1 | low |
| TCGA-AA-3526 | 57 | MALE | Stage I | T2 | M0 | N0 | low |
| TCGA-AA-3527 | 90 | FEMALE | Stage II | T3 | M0 | N0 | low |
| TCGA-AA-3529 | 78 | FEMALE | Stage III | T3 | M0 | N2 | low |
| TCGA-AA-3530 | 80 | MALE | Stage I | T2 | M0 | N0 | high |
| TCGA-AA-3531 | 75 | FEMALE | Stage II | T3 | M0 | N0 | high |
| TCGA-AA-3532 | 63 | MALE | Stage II | T3 | M0 | N0 | low |
| TCGA-AA-3534 | 78 | FEMALE | Stage II | T3 | M0 | N0 | low |
| TCGA-AA-3538 | 54 | FEMALE | Stage I | T2 | M0 | N0 | high |
| TCGA-AA-3542 | 69 | MALE | Stage III | T3 | M0 | N2 | high |
| TCGA-AA-3543 | 84 | MALE | Stage I | T2 | M0 | N0 | high |
| TCGA-AA-3544 | 68 | MALE | Stage I | T2 | M0 | N0 | low |
| TCGA-AA-3548 | 71 | FEMALE | Stage III | T3 | M0 | N2 | high |
| TCGA-AA-3549 | 69 | MALE | Stage I | T2 | M0 | N0 | high |
| TCGA-AA-3552 | 85 | MALE | Stage III | T3 | M0 | N2 | high |
| TCGA-AA-3553 | 61 | FEMALE | Stage I | T2 | M0 | N0 | low |
| TCGA-AA-3554 | 62 | FEMALE | Stage II | T3 | M0 | N0 | high |
| TCGA-AA-3555 | 81 | FEMALE | Stage II | T3 | M0 | N0 | high |
| TCGA-AA-3556 | 78 | MALE | Stage I | T2 | M0 | N0 | low |
| TCGA-AA-3560 | 72 | FEMALE | Stage III | T3 | M0 | N2 | low |
| TCGA-AA-3561 | 72 | MALE | Stage II | T3 | M0 | N0 | low |
| TCGA-AA-3562 | 82 | MALE | Stage III | T3 | M0 | N2 | high |
| TCGA-AA-3655 | 68 | MALE | Stage II | T3 | M0 | N0 | low |
| TCGA-AA-3660 | 51 | FEMALE | Stage II | T3 | M0 | N0 | high |
| TCGA-AA-3662 | 80 | FEMALE | Stage IV | T4 | M1 | N2 | high |
| TCGA-AA-3663 | 42 | MALE | Stage II | T3 | M0 | N0 | low |
| TCGA-AA-3664 | 74 | FEMALE | Stage II | T3 | M0 | N0 | high |
| TCGA-AA-3666 | 68 | MALE | Stage III | T3 | M0 | N1 | low |
| TCGA-AA-3667 | 36 | FEMALE | Stage I | T2 | M0 | N0 | low |
| TCGA-AA-3672 | 90 | FEMALE | Stage III | T3 | M0 | N1 | high |
| TCGA-AA-3673 | 53 | FEMALE | Stage II | T3 | M0 | N0 | low |
| TCGA-AA-3675 | 84 | MALE | Stage II | T3 | M0 | N0 | low |
| TCGA-AA-3678 | 60 | FEMALE | Stage III | T2 | M0 | N1 | low |
| TCGA-AA-3679 | 59 | MALE | Stage IV | T3 | M1 | N2 | low |
| TCGA-AA-3680 | 67 | FEMALE | Stage IV | T4 | M1 | N2 | high |
| TCGA-AA-3681 | 77 | FEMALE | Stage III | T3 | M0 | N1 | high |
| TCGA-AA-3684 | 65 | FEMALE | Stage IV | T4 | M1 | N2 | high |
| TCGA-AA-3685 | 69 | MALE | Stage II | T3 | M0 | N0 | low |
| TCGA-AA-3688 | 80 | MALE | Stage IV | T3 | M1 | N1 | low |
| TCGA-AA-3692 | 47 | FEMALE | Stage IV | T3 | M1 | N2 | high |
| TCGA-AA-3693 | 77 | FEMALE | Stage IV | T4 | M1 | N1 | low |
| TCGA-AA-3696 | 75 | FEMALE | Stage IV | T3 | M1 | N1 | high |
| TCGA-AA-3697 | 77 | MALE | Stage II | T3 | M0 | N0 | low |
| TCGA-AA-3710 | 80 | FEMALE | Stage II | T3 | M0 | N0 | high |
| TCGA-AA-3712 | 65 | MALE | Stage III | T3 | M0 | N2 | low |
| TCGA-AA-3713 | 68 | MALE | Stage IV | T3 | M1 | N0 | low |
| TCGA-AA-3715 | 77 | MALE | Stage II | T3 | M0 | N0 | high |
| TCGA-AA-3811 | 84 | FEMALE | Stage III | T3 | M0 | N2 | high |
| TCGA-AA-3812 | 82 | FEMALE | Stage II | T3 | M0 | N0 | high |
| TCGA-AA-3814 | 85 | FEMALE | Stage II | T3 | M0 | N0 | low |
| TCGA-AA-3815 | 65 | FEMALE | Stage II | T3 | M0 | N0 | low |
| TCGA-AA-3818 | 78 | FEMALE | Stage II | T3 | M0 | N0 | low |
| TCGA-AA-3819 | 41 | FEMALE | Stage II | T3 | M0 | N0 | low |
| TCGA-AA-3821 | 81 | FEMALE | Stage I | T2 | M0 | N0 | low |
| TCGA-AA-3831 | 66 | MALE | Stage II | T3 | M0 | N0 | low |
| TCGA-AA-3833 | 63 | FEMALE | Stage II | T3 | M0 | N0 | high |
| TCGA-AA-3837 | 67 | MALE | Stage II | T3 | M0 | N0 | high |
| TCGA-AA-3841 | 66 | MALE | Stage II | T3 | M0 | N0 | high |
| TCGA-AA-3842 | 51 | MALE | Stage III | T2 | M0 | N1 | low |
| TCGA-AA-3844 | 78 | FEMALE | Stage III | T3 | M0 | N2 | low |
| TCGA-AA-3845 | 86 | FEMALE | Stage II | T3 | M0 | N0 | high |
| TCGA-AA-3846 | 74 | FEMALE | Stage II | T3 | M0 | N0 | low |
| TCGA-AA-3848 | 82 | FEMALE | Stage III | T3 | M0 | N2 | high |
| TCGA-AA-3850 | 74 | MALE | Stage I | T2 | M0 | N0 | low |
| TCGA-AA-3851 | 74 | MALE | Stage II | T3 | M0 | N0 | high |
| TCGA-AA-3852 | 88 | MALE | Stage II | T3 | M0 | N0 | low |
| TCGA-AA-3854 | 71 | FEMALE | Stage I | T2 | M0 | N0 | low |
| TCGA-AA-3855 | 72 | MALE | Stage I | T2 | M0 | N0 | low |
| TCGA-AA-3856 | 59 | MALE | Stage II | T3 | M0 | N0 | low |
| TCGA-AA-3858 | 67 | MALE | Stage I | T2 | M0 | N0 | low |
| TCGA-AA-3860 | 53 | FEMALE | Stage III | T3 | M0 | N1 | high |
| TCGA-AA-3861 | 72 | MALE | Stage II | T3 | M0 | N0 | low |
| TCGA-AA-3862 | 82 | MALE | Stage II | T3 | M0 | N0 | low |
| TCGA-AA-3864 | 71 | MALE | Stage II | T3 | M0 | N0 | low |
| TCGA-AA-3866 | 78 | FEMALE | Stage I | T2 | M0 | N0 | low |
| TCGA-AA-3867 | 74 | MALE | Stage IV | T3 | M1 | N2 | high |
| TCGA-AA-3869 | 76 | MALE | Stage IV | T4 | M1 | N2 | low |
| TCGA-AA-3870 | 71 | FEMALE | Stage IV | T3 | M1 | N2 | high |
| TCGA-AA-3872 | 45 | MALE | Stage IV | T4 | M1 | N2 | high |
| TCGA-AA-3875 | 78 | FEMALE | Stage I | T1 | M0 | N0 | low |
| TCGA-AA-3877 | 83 | FEMALE | Stage I | T1 | M0 | N0 | high |
| TCGA-AA-3930 | 66 | MALE | Stage IV | T3 | M1 | N2 | high |
| TCGA-AA-3939 | 83 | MALE | Stage II | T3 | M0 | N0 | low |
| TCGA-AA-3941 | 84 | FEMALE | Stage IV | T4 | M1 | N1 | low |
| TCGA-AA-3947 | 60 | FEMALE | Stage II | T4 | M0 | N0 | low |
| TCGA-AA-3949 | 87 | FEMALE | Stage III | T3 | M0 | N1 | low |
| TCGA-AA-3950 | 79 | FEMALE | Stage II | T3 | M0 | N0 | high |
| TCGA-AA-3952 | 68 | MALE | Stage III | T3 | M0 | N2 | high |
| TCGA-AA-3955 | 38 | MALE | Stage III | T2 | M0 | N2 | low |
| TCGA-AA-3956 | 65 | MALE | Stage II | T3 | M0 | N0 | low |
| TCGA-AA-3966 | 89 | FEMALE | Stage II | T3 | M0 | N0 | high |
| TCGA-AA-3968 | 55 | FEMALE | Stage I | T2 | M0 | N0 | low |
| TCGA-AA-3970 | 65 | MALE | Stage II | T3 | M0 | N0 | low |
| TCGA-AA-3971 | 58 | MALE | Stage III | T3 | M0 | N1 | high |
| TCGA-AA-3972 | 72 | MALE | Stage IV | T3 | M1 | N1 | low |
| TCGA-AA-3973 | 69 | MALE | Stage IV | T4 | M1 | N1 | high |
| TCGA-AA-3975 | 80 | MALE | Stage I | T2 | M0 | N0 | high |
| TCGA-AA-3977 | 65 | MALE | unknow | T2 | unknow | N0 | low |
| TCGA-AA-3979 | 84 | MALE | Stage II | T3 | M0 | N0 | low |
| TCGA-AA-3980 | 89 | FEMALE | Stage I | T2 | M0 | N0 | high |
| TCGA-AA-3982 | 75 | MALE | Stage III | T3 | M0 | N1 | high |
| TCGA-AA-3984 | 61 | FEMALE | Stage II | T3 | M0 | N0 | low |
| TCGA-AA-3986 | 73 | MALE | Stage I | T2 | M0 | N0 | low |
| TCGA-AA-3989 | 84 | MALE | Stage IV | T3 | M1 | N2 | low |
| TCGA-AA-3994 | 69 | MALE | Stage III | T3 | M0 | N1 | low |
| TCGA-AA-A004 | 76 | MALE | Stage II | T3 | M0 | N0 | high |
| TCGA-AA-A00A | 80 | MALE | Stage II | T3 | M0 | N0 | high |
| TCGA-AA-A00D | 70 | MALE | Stage I | T2 | M0 | N0 | low |
| TCGA-AA-A00E | 65 | MALE | Stage II | T3 | M0 | N0 | high |
| TCGA-AA-A00F | 66 | MALE | Stage III | T3 | M0 | N2 | high |
| TCGA-AA-A00J | 80 | MALE | Stage III | T4 | M0 | N1 | high |
| TCGA-AA-A00K | 79 | MALE | Stage II | T3 | M0 | N0 | high |
| TCGA-AA-A00L | 66 | MALE | Stage II | T3 | M0 | N0 | low |
| TCGA-AA-A00N | 75 | MALE | Stage II | T4 | M0 | N0 | high |
| TCGA-AA-A00O | 83 | FEMALE | Stage III | T3 | M0 | N2 | high |
| TCGA-AA-A00Q | 66 | FEMALE | Stage III | T4 | M0 | N1 | low |
| TCGA-AA-A00R | 64 | FEMALE | Stage I | T2 | M0 | N0 | low |
| TCGA-AA-A00U | 50 | MALE | Stage III | T3 | M0 | N1 | high |
| TCGA-AA-A00W | 80 | MALE | Stage I | T1 | M0 | N0 | low |
| TCGA-AA-A00Z | 70 | MALE | Stage II | T3 | M0 | N0 | low |
| TCGA-AA-A010 | 46 | FEMALE | Stage II | T4 | M0 | N0 | high |
| TCGA-AA-A017 | 57 | FEMALE | Stage II | T3 | M0 | N0 | high |
| TCGA-AA-A01C | 75 | MALE | Stage III | T2 | M0 | N1 | high |
| TCGA-AA-A01F | 72 | MALE | Stage III | T3 | M0 | N1 | low |
| TCGA-AA-A01G | 63 | MALE | Stage II | T3 | M0 | N0 | low |
| TCGA-AA-A01I | 76 | MALE | Stage I | T2 | M0 | N0 | low |
| TCGA-AA-A01K | 74 | FEMALE | Stage III | T3 | M0 | N2 | high |
| TCGA-AA-A01P | 80 | FEMALE | Stage III | T3 | M0 | N1 | high |
| TCGA-AA-A01Q | 48 | FEMALE | Stage II | T3 | M0 | N0 | high |
| TCGA-AA-A01R | 47 | MALE | Stage III | T3 | M0 | N2 | high |
| TCGA-AA-A01S | 47 | FEMALE | Stage III | T3 | M0 | N1 | low |
| TCGA-AA-A01T | 63 | FEMALE | Stage III | T3 | M0 | N1 | high |
| TCGA-AA-A01V | 59 | MALE | Stage I | T2 | M0 | N0 | high |
| TCGA-AA-A01X | 80 | FEMALE | Stage III | T2 | M0 | N1 | high |
| TCGA-AA-A01Z | 68 | MALE | Stage II | T3 | M0 | N0 | high |
| TCGA-AA-A022 | 88 | FEMALE | Stage II | T4 | M0 | N0 | high |
| TCGA-AA-A024 | 81 | MALE | Stage II | T3 | M0 | N0 | high |
| TCGA-AA-A029 | 67 | MALE | Stage II | T3 | M0 | N0 | high |
| TCGA-AA-A02E | 82 | FEMALE | Stage IV | T3 | M1 | N1 | high |
| TCGA-AA-A02F | 68 | FEMALE | Stage IV | T3 | M1 | N1 | high |
| TCGA-AA-A02H | 74 | FEMALE | Stage IV | T3 | M1 | N2 | high |
| TCGA-AA-A02J | 70 | FEMALE | Stage IV | T3 | M1 | N0 | high |
| TCGA-AA-A02K | 50 | MALE | Stage IV | T4 | M1 | N2 | high |
| TCGA-AA-A02O | 82 | MALE | Stage II | T3 | M0 | N0 | high |
| TCGA-AA-A02R | 84 | FEMALE | Stage II | T3 | M0 | N0 | high |
| TCGA-AA-A02W | 73 | FEMALE | Stage I | T2 | M0 | N0 | high |
| TCGA-AA-A02Y | 73 | MALE | Stage I | T2 | M0 | N0 | low |
| TCGA-AA-A03F | 90 | FEMALE | Stage III | T3 | M0 | N2 | high |
| TCGA-AA-A03J | 65 | FEMALE | Stage I | T2 | M0 | N0 | low |
| TCGA-AD-5900 | 67 | MALE | Stage I | T2 | unknow | N0 | low |
| TCGA-AD-6548 | 81 | FEMALE | Stage I | T2 | M0 | N0 | low |
| TCGA-AD-6888 | 73 | MALE | Stage III | T3 | M0 | N1 | high |
| TCGA-AD-6889 | 76 | MALE | Stage II | T3 | M0 | N0 | high |
| TCGA-AD-6890 | 65 | MALE | unknow | T1 | unknow | N0 | low |
| TCGA-AD-6895 | 84 | MALE | Stage III | T3 | M0 | N1 | high |
| TCGA-AD-6899 | 84 | MALE | Stage III | T4 | unknow | N2 | high |
| TCGA-AD-6901 | 78 | MALE | unknow | T3 | unknow | N0 | high |
| TCGA-AD-6963 | 58 | MALE | unknow | T3 | unknow | N0 | low |
| TCGA-AD-6964 | 58 | MALE | unknow | T4 | unknow | N2 | high |
| TCGA-AD-6965 | 62 | MALE | Stage III | T4 | M0 | N2 | low |
| TCGA-AD-A5EJ | 74 | FEMALE | Stage II | T3 | unknow | N0 | high |
| TCGA-AD-A5EK | 51 | MALE | Stage I | T2 | unknow | N0 | high |
| TCGA-AF-2690 | 76 | FEMALE | Stage III | T3 | M0 | N2 | high |
| TCGA-AF-2692 | 54 | FEMALE | Stage II | T3 | M0 | N0 | high |
| TCGA-AF-3911 | 48 | MALE | Stage III | T3 | unknow | N2 | high |
| TCGA-AF-4110 | 77 | MALE | Stage IV | T4 | unknow | N2 | high |
| TCGA-AG-3574 | 89 | FEMALE | Stage II | T3 | M0 | N0 | high |
| TCGA-AG-3575 | 51 | MALE | Stage II | T3 | M0 | N0 | high |
| TCGA-AG-3582 | 75 | MALE | Stage IV | T3 | M1 | N1 | high |
| TCGA-AG-3584 | 60 | MALE | Stage IV | T3 | M1 | N2 | high |
| TCGA-AG-3587 | 65 | MALE | Stage I | T2 | M0 | N0 | high |
| TCGA-AG-3608 | 79 | FEMALE | Stage II | T3 | M0 | N0 | high |
| TCGA-AG-3612 | 55 | FEMALE | Stage III | T3 | M0 | N1 | high |
| TCGA-AG-3726 | 63 | FEMALE | Stage I | T2 | M0 | N0 | low |
| TCGA-AG-3728 | 73 | MALE | Stage III | T3 | M0 | N1 | high |
| TCGA-AG-3731 | 65 | MALE | Stage IV | T3 | M1 | N1 | low |
| TCGA-AG-3742 | 85 | FEMALE | Stage I | T1 | M0 | N0 | high |
| TCGA-AG-3878 | 64 | MALE | Stage I | T2 | M0 | N0 | high |
| TCGA-AG-3882 | 66 | FEMALE | Stage I | T2 | M0 | N0 | high |
| TCGA-AG-3883 | 69 | MALE | Stage I | T2 | M0 | N0 | high |
| TCGA-AG-3885 | 71 | FEMALE | Stage III | T3 | M0 | N1 | low |
| TCGA-AG-3887 | 68 | MALE | Stage II | T3 | M0 | N0 | low |
| TCGA-AG-3890 | 62 | MALE | Stage I | T2 | M0 | N0 | high |
| TCGA-AG-3892 | 57 | FEMALE | Stage I | T1 | M0 | N0 | low |
| TCGA-AG-3894 | 65 | MALE | Stage II | T3 | M0 | N0 | high |
| TCGA-AG-3896 | 85 | FEMALE | Stage I | T2 | M0 | N0 | low |
| TCGA-AG-3898 | 61 | MALE | Stage II | T3 | M0 | N0 | high |
| TCGA-AG-4005 | 64 | MALE | Stage IV | T3 | M1 | N2 | high |
| TCGA-AG-4007 | 87 | MALE | Stage IV | T4 | M1 | N2 | high |
| TCGA-AG-4008 | 63 | MALE | Stage II | T3 | M0 | N0 | high |
| TCGA-AG-4015 | 85 | FEMALE | Stage II | T3 | M0 | N0 | low |
| TCGA-AG-4021 | 84 | FEMALE | Stage IV | T3 | M1 | N2 | high |
| TCGA-AG-4022 | 59 | FEMALE | Stage II | T3 | M0 | N0 | low |
| TCGA-AG-A002 | 35 | MALE | Stage I | T2 | M0 | N0 | high |
| TCGA-AG-A008 | 50 | FEMALE | Stage I | T2 | M0 | N0 | low |
| TCGA-AG-A00C | 49 | FEMALE | Stage III | T3 | M0 | N1 | high |
| TCGA-AG-A00H | 75 | MALE | Stage II | T3 | M0 | N0 | high |
| TCGA-AG-A00Y | 68 | MALE | Stage II | T3 | M0 | N0 | high |
| TCGA-AG-A011 | 80 | MALE | Stage II | T3 | M0 | N0 | high |
| TCGA-AG-A014 | 86 | MALE | Stage I | T2 | M0 | N0 | low |
| TCGA-AG-A015 | 64 | FEMALE | Stage I | T1 | M0 | N0 | low |
| TCGA-AG-A016 | 55 | MALE | Stage IV | T3 | M1 | N2 | low |
| TCGA-AG-A01J | 59 | FEMALE | Stage II | T3 | M0 | N0 | high |
| TCGA-AG-A01L | 58 | MALE | Stage III | T3 | M0 | N1 | high |
| TCGA-AG-A01N | 68 | FEMALE | Stage IV | T2 | M1 | N0 | low |
| TCGA-AG-A01W | 67 | FEMALE | Stage II | T3 | M0 | N0 | high |
| TCGA-AG-A01Y | 49 | FEMALE | Stage II | T3 | M0 | N0 | high |
| TCGA-AG-A020 | 57 | FEMALE | Stage III | T3 | M0 | N1 | low |
| TCGA-AG-A023 | 62 | FEMALE | Stage IV | T4 | M1 | N2 | high |
| TCGA-AG-A025 | 62 | FEMALE | Stage I | T1 | M0 | N0 | low |
| TCGA-AG-A026 | 66 | MALE | Stage II | T4 | M0 | N0 | high |
| TCGA-AG-A02G | 66 | MALE | Stage IV | T2 | M1 | N1 | high |
| TCGA-AG-A02N | 67 | MALE | Stage II | T3 | M0 | N0 | high |
| TCGA-AG-A02X | 77 | MALE | Stage I | T2 | M0 | N0 | low |
| TCGA-AG-A032 | 68 | MALE | Stage III | T3 | M0 | N1 | low |
| TCGA-AG-A036 | 71 | MALE | Stage III | T3 | M0 | N2 | low |
| TCGA-AH-6549 | 66 | MALE | unknow | T3 | unknow | unknow | low |
| TCGA-AM-5820 | 59 | FEMALE | Stage IV | T4 | M1 | N2 | low |
| TCGA-AM-5821 | 68 | FEMALE | Stage II | T3 | M0 | N0 | high |
| TCGA-AU-3779 | 80 | FEMALE | Stage II | T3 | M0 | N0 | low |
| TCGA-AU-6004 | 69 | FEMALE | Stage I | T2 | M0 | N0 | low |
| TCGA-AY-4070 | 50 | FEMALE | Stage III | T3 | M0 | N2 | high |
| TCGA-AY-4071 | 63 | FEMALE | Stage I | T1 | unknow | N0 | high |
| TCGA-AY-5543 | 65 | FEMALE | Stage IV | T3 | M1 | N1 | low |
| TCGA-AY-6196 | 47 | MALE | Stage III | T3 | unknow | N2 | high |
| TCGA-AY-6197 | 60 | MALE | Stage II | T3 | unknow | N0 | low |
| TCGA-AY-6386 | 66 | FEMALE | Stage III | T3 | M0 | N1 | low |
| TCGA-AY-A54L | 74 | FEMALE | Stage I | T2 | M0 | N0 | low |
| TCGA-AY-A69D | 55 | FEMALE | Stage II | T3 | M0 | N0 | low |
| TCGA-AY-A71X | 54 | FEMALE | Stage I | T2 | M0 | N0 | low |
| TCGA-AY-A8YK | 44 | MALE | Stage IV | T3 | M1 | N2 | low |
| TCGA-AZ-4308 | 47 | FEMALE | Stage III | T3 | M0 | N1 | high |
| TCGA-AZ-4313 | 51 | FEMALE | Stage I | T1 | M0 | N0 | low |
| TCGA-AZ-4315 | 61 | MALE | Stage II | T3 | M0 | N0 | low |
| TCGA-AZ-4323 | 37 | MALE | Stage IV | T4 | M1 | N2 | high |
| TCGA-AZ-4614 | 71 | FEMALE | Stage IV | T4 | M1 | N1 | high |
| TCGA-AZ-4615 | 84 | MALE | Stage III | T3 | M0 | N1 | high |
| TCGA-AZ-4616 | 82 | FEMALE | Stage IV | T3 | M1 | N2 | high |
| TCGA-AZ-5403 | 43 | MALE | Stage II | T3 | unknow | N0 | low |
| TCGA-AZ-5407 | 51 | FEMALE | Stage I | T1 | M0 | N0 | low |
| TCGA-AZ-6598 | 77 | FEMALE | Stage II | T3 | unknow | N0 | low |
| TCGA-AZ-6599 | 72 | MALE | Stage I | T2 | unknow | N0 | low |
| TCGA-AZ-6600 | 64 | MALE | Stage IV | T4 | M1 | N1 | high |
| TCGA-AZ-6601 | 68 | MALE | Stage II | T3 | M0 | N0 | low |
| TCGA-AZ-6603 | 77 | FEMALE | unknow | T2 | unknow | N1 | low |
| TCGA-AZ-6605 | 77 | MALE | Stage III | T4 | M0 | N1 | low |
| TCGA-AZ-6606 | 81 | MALE | Stage IV | T4 | M1 | N2 | low |
| TCGA-AZ-6607 | 69 | MALE | Stage IV | T4 | M1 | N2 | high |
| TCGA-AZ-6608 | 55 | FEMALE | Stage III | T2 | M0 | N1 | low |
| TCGA-BM-6198 | 73 | MALE | Stage III | T3 | unknow | N1 | high |
| TCGA-CA-5254 | 42 | FEMALE | Stage II | T3 | M0 | N0 | low |
| TCGA-CA-5255 | 45 | MALE | Stage II | T3 | M0 | N0 | low |
| TCGA-CA-5256 | 54 | FEMALE | Stage II | T3 | M0 | N0 | low |
| TCGA-CA-5796 | 52 | FEMALE | Stage II | T3 | M0 | N0 | low |
| TCGA-CA-5797 | 56 | MALE | Stage II | T3 | M0 | N0 | low |
| TCGA-CA-6715 | 63 | MALE | Stage III | T3 | M0 | N1 | high |
| TCGA-CA-6716 | 65 | MALE | Stage II | T3 | M0 | N0 | high |
| TCGA-CA-6717 | 57 | MALE | Stage II | T3 | M0 | N0 | high |
| TCGA-CA-6718 | 46 | MALE | Stage II | T3 | M0 | N0 | high |
| TCGA-CA-6719 | 77 | MALE | Stage II | T3 | M0 | N0 | high |
| TCGA-CI-6619 | 41 | MALE | Stage IV | T3 | M1 | N1 | low |
| TCGA-CI-6620 | 41 | FEMALE | Stage IV | T3 | M1 | N1 | low |
| TCGA-CI-6621 | 63 | MALE | Stage III | T3 | unknow | N1 | high |
| TCGA-CI-6622 | 74 | MALE | Stage II | T4 | M0 | N0 | low |
| TCGA-CI-6623 | 44 | MALE | Stage I | T1 | M0 | N0 | low |
| TCGA-CI-6624 | 53 | FEMALE | Stage I | T2 | M0 | N0 | low |
| TCGA-CK-4947 | 46 | FEMALE | Stage III | T4 | M0 | N1 | low |
| TCGA-CK-4948 | 45 | FEMALE | Stage III | T3 | M0 | N1 | low |
| TCGA-CK-4950 | 68 | FEMALE | Stage III | T3 | M0 | N1 | low |
| TCGA-CK-4951 | 79 | FEMALE | Stage II | T3 | M0 | N0 | low |
| TCGA-CK-4952 | 48 | FEMALE | Stage III | T4 | M0 | N2 | low |
| TCGA-CK-5912 | 81 | MALE | Stage I | T2 | unknow | N0 | low |
| TCGA-CK-5913 | 58 | FEMALE | Stage II | T3 | unknow | N0 | low |
| TCGA-CK-5914 | 81 | MALE | Stage III | T3 | unknow | N1 | low |
| TCGA-CK-5915 | 63 | MALE | Stage I | T2 | unknow | N0 | low |
| TCGA-CK-5916 | 71 | FEMALE | Stage I | T1 | M0 | N0 | high |
| TCGA-CK-6746 | 84 | FEMALE | Stage II | T4 | unknow | N0 | high |
| TCGA-CK-6747 | 87 | FEMALE | Stage II | T3 | unknow | N0 | low |
| TCGA-CK-6748 | 45 | FEMALE | Stage IV | T3 | M1 | N1 | low |
| TCGA-CK-6751 | 88 | FEMALE | Stage I | T2 | unknow | N0 | low |
| TCGA-CL-4957 | 79 | FEMALE | unknow | T3 | M0 | N1 | low |
| TCGA-CL-5918 | 90 | FEMALE | Stage II | T3 | unknow | N0 | low |
| TCGA-CM-4743 | 69 | MALE | Stage II | T3 | M0 | N0 | high |
| TCGA-CM-4744 | 69 | MALE | Stage I | T2 | M0 | N0 | low |
| TCGA-CM-4746 | 61 | MALE | Stage I | T2 | M0 | N0 | low |
| TCGA-CM-4747 | 47 | MALE | Stage IV | T4 | M1 | N1 | high |
| TCGA-CM-4748 | 53 | MALE | Stage III | T4 | M0 | N1 | low |
| TCGA-CM-4751 | 62 | MALE | Stage III | T3 | M0 | N1 | high |
| TCGA-CM-4752 | 58 | MALE | Stage II | T3 | M0 | N0 | high |
| TCGA-CM-5341 | 82 | FEMALE | Stage III | T2 | M0 | N1 | low |
| TCGA-CM-5344 | 39 | FEMALE | Stage III | T3 | M0 | N1 | high |
| TCGA-CM-5348 | 72 | MALE | Stage III | T3 | M0 | N1 | high |
| TCGA-CM-5349 | 68 | FEMALE | Stage II | T3 | M0 | N0 | low |
| TCGA-CM-5860 | 44 | MALE | Stage II | T3 | M0 | N0 | low |
| TCGA-CM-5861 | 63 | FEMALE | Stage II | T3 | M0 | N0 | low |
| TCGA-CM-5862 | 80 | MALE | Stage IV | T3 | M1 | N1 | high |
| TCGA-CM-5863 | 60 | FEMALE | Stage III | T3 | M0 | N1 | high |
| TCGA-CM-5864 | 60 | MALE | Stage I | T2 | M0 | N0 | low |
| TCGA-CM-5868 | 59 | FEMALE | Stage IV | T4 | M1 | N1 | high |
| TCGA-CM-6161 | 36 | FEMALE | Stage I | T2 | M0 | N0 | low |
| TCGA-CM-6162 | 48 | FEMALE | Stage III | T3 | M0 | N1 | low |
| TCGA-CM-6163 | 74 | MALE | Stage I | T1 | M0 | N0 | high |
| TCGA-CM-6164 | 46 | FEMALE | Stage II | T3 | M0 | N0 | low |
| TCGA-CM-6165 | 74 | MALE | Stage II | T3 | M0 | N0 | low |
| TCGA-CM-6166 | 48 | FEMALE | Stage I | T2 | M0 | N0 | high |
| TCGA-CM-6167 | 57 | FEMALE | Stage III | T3 | M0 | N2 | high |
| TCGA-CM-6168 | 84 | FEMALE | Stage II | T3 | M0 | N0 | low |
| TCGA-CM-6169 | 67 | MALE | Stage II | T3 | M0 | N0 | high |
| TCGA-CM-6170 | 73 | FEMALE | Stage I | T2 | M0 | N0 | low |
| TCGA-CM-6171 | 77 | FEMALE | Stage I | T2 | M0 | N0 | low |
| TCGA-CM-6172 | 70 | FEMALE | Stage III | T3 | M0 | N1 | low |
| TCGA-CM-6674 | 39 | MALE | Stage II | T3 | M0 | N0 | low |
| TCGA-CM-6675 | 35 | MALE | Stage IV | T3 | M1 | N2 | high |
| TCGA-CM-6676 | 82 | MALE | Stage I | T2 | M0 | N0 | high |
| TCGA-CM-6677 | 75 | FEMALE | Stage II | T3 | M0 | N0 | low |
| TCGA-CM-6678 | 63 | FEMALE | Stage IV | T4 | M1 | N1 | high |
| TCGA-CM-6679 | 58 | MALE | Stage II | T3 | M0 | N0 | high |
| TCGA-CM-6680 | 78 | FEMALE | Stage III | T3 | M0 | N2 | high |
| TCGA-D5-5537 | 83 | MALE | unknow | T3 | unknow | N2 | high |
| TCGA-D5-5538 | 60 | FEMALE | Stage III | T3 | M0 | N1 | high |
| TCGA-D5-5539 | 60 | MALE | Stage III | T3 | M0 | N1 | high |
| TCGA-D5-5540 | 73 | MALE | Stage II | T3 | M0 | N0 | low |
| TCGA-D5-5541 | 63 | MALE | Stage III | T3 | M0 | N1 | high |
| TCGA-D5-6529 | 69 | MALE | Stage II | T3 | M0 | N0 | low |
| TCGA-D5-6530 | 53 | MALE | Stage I | T2 | M0 | N0 | low |
| TCGA-D5-6531 | 75 | MALE | Stage II | T3 | M0 | N0 | high |
| TCGA-D5-6532 | 61 | MALE | Stage II | T3 | M0 | N0 | low |
| TCGA-D5-6533 | 68 | FEMALE | unknow | T4 | M0 | N0 | low |
| TCGA-D5-6534 | 62 | FEMALE | Stage II | T3 | M0 | N0 | high |
| TCGA-D5-6535 | 80 | FEMALE | Stage III | T3 | unknow | N1 | high |
| TCGA-D5-6536 | 73 | MALE | Stage II | T3 | M0 | N0 | high |
| TCGA-D5-6537 | 64 | MALE | Stage III | T3 | unknow | N1 | low |
| TCGA-D5-6538 | 79 | FEMALE | Stage III | T3 | M0 | N2 | low |
| TCGA-D5-6539 | 45 | FEMALE | unknow | T3 | M0 | N0 | low |
| TCGA-D5-6540 | 66 | MALE | Stage I | T2 | M0 | N0 | high |
| TCGA-D5-6541 | 49 | MALE | Stage II | T3 | M0 | N0 | low |
| TCGA-D5-6898 | 51 | FEMALE | Stage I | T2 | M0 | N0 | low |
| TCGA-D5-6920 | 77 | FEMALE | Stage II | T3 | M0 | N0 | low |
| TCGA-D5-6922 | 76 | MALE | Stage III | T3 | M0 | N1 | high |
| TCGA-D5-6923 | 57 | MALE | Stage I | T2 | M0 | N0 | high |
| TCGA-D5-6924 | 68 | MALE | Stage II | T3 | M0 | N0 | high |
| TCGA-D5-6926 | 65 | MALE | Stage III | T4 | M0 | N1 | high |
| TCGA-D5-6927 | 34 | MALE | Stage II | T3 | M0 | N0 | high |
| TCGA-D5-6928 | 80 | MALE | Stage II | T3 | M0 | N0 | high |
| TCGA-D5-6929 | 49 | FEMALE | Stage IV | T3 | M1 | N1 | high |
| TCGA-D5-6930 | 67 | MALE | Stage II | T3 | M0 | N0 | low |
| TCGA-D5-6931 | 77 | MALE | Stage III | T4 | M0 | N2 | low |
| TCGA-D5-6932 | 69 | MALE | Stage II | T3 | M0 | N0 | high |
| TCGA-D5-7000 | 79 | FEMALE | Stage I | T2 | M0 | N0 | low |
| TCGA-DC-5869 | 62 | FEMALE | Stage III | T3 | M0 | N1 | high |
| TCGA-DC-6158 | 70 | MALE | Stage I | T2 | M0 | N0 | high |
| TCGA-DM-A0X9 | 71 | FEMALE | Stage II | T3 | M0 | N0 | low |
| TCGA-DM-A0XD | 65 | MALE | Stage II | T3 | M0 | N0 | high |
| TCGA-DM-A0XF | 68 | FEMALE | Stage III | T3 | M0 | N2 | low |
| TCGA-DM-A1D0 | 79 | FEMALE | Stage II | T3 | M0 | N0 | low |
| TCGA-DM-A1D4 | 80 | MALE | Stage II | T3 | M0 | N0 | low |
| TCGA-DM-A1D6 | 88 | MALE | Stage II | T3 | M0 | N0 | low |
| TCGA-DM-A1D7 | 82 | MALE | Stage II | T3 | M0 | N0 | low |
| TCGA-DM-A1D8 | 50 | FEMALE | unknow | T3 | unknow | N1 | high |
| TCGA-DM-A1D9 | 67 | FEMALE | Stage II | T3 | M0 | N0 | low |
| TCGA-DM-A1DA | 71 | FEMALE | Stage III | T3 | M0 | N2 | high |
| TCGA-DM-A1DB | 68 | MALE | Stage II | T3 | M0 | N0 | high |
| TCGA-DM-A1HA | 82 | MALE | Stage III | T3 | M0 | N2 | low |
| TCGA-DM-A1HB | 75 | MALE | Stage III | T3 | M0 | N1 | low |
| TCGA-DM-A280 | 70 | FEMALE | Stage II | T3 | M0 | N0 | low |
| TCGA-DM-A282 | 60 | FEMALE | Stage II | T3 | M0 | N0 | high |
| TCGA-DM-A285 | 71 | FEMALE | Stage IV | T3 | M1 | N2 | high |
| TCGA-DM-A288 | 68 | MALE | Stage III | T3 | M0 | N2 | high |
| TCGA-DM-A28A | 78 | MALE | Stage III | T3 | M0 | N2 | high |
| TCGA-DM-A28C | 74 | MALE | Stage II | T3 | M0 | N0 | low |
| TCGA-DM-A28E | 72 | FEMALE | Stage II | T3 | M0 | N0 | low |
| TCGA-DM-A28F | 73 | MALE | Stage III | T3 | M0 | N1 | low |
| TCGA-DM-A28G | 75 | MALE | Stage II | T3 | M0 | N0 | high |
| TCGA-DM-A28H | 50 | MALE | Stage III | T3 | M0 | N2 | low |
| TCGA-DM-A28K | 75 | MALE | Stage II | T3 | M0 | N0 | low |
| TCGA-DM-A28M | 63 | MALE | Stage II | T3 | M0 | N0 | low |
| TCGA-DT-5265 | 51 | MALE | Stage II | T3 | M0 | N0 | high |
| TCGA-DY-A1DE | 56 | FEMALE | Stage II | T3 | M0 | N0 | low |
| TCGA-DY-A1DG | 75 | MALE | Stage IV | T3 | M1 | N1 | high |
| TCGA-DY-A1H8 | 77 | FEMALE | Stage III | T2 | M0 | N1 | high |
| TCGA-EF-5830 | 54 | MALE | Stage II | T4 | M0 | N0 | low |
| TCGA-EF-5831 | 72 | MALE | Stage II | T3 | M0 | N0 | low |
| TCGA-EI-6506 | 78 | FEMALE | Stage II | T3 | M0 | N0 | low |
| TCGA-EI-6507 | 60 | MALE | Stage II | T3 | M0 | N0 | high |
| TCGA-EI-6508 | 48 | FEMALE | Stage III | T3 | M0 | N1 | low |
| TCGA-EI-6509 | 53 | MALE | Stage III | T3 | M0 | N2 | low |
| TCGA-EI-6510 | 77 | FEMALE | unknow | T2 | unknow | N0 | low |
| TCGA-EI-6511 | 52 | MALE | Stage III | T3 | M0 | N1 | low |
| TCGA-EI-6512 | 64 | FEMALE | Stage III | T3 | M0 | N1 | low |
| TCGA-EI-6513 | 59 | MALE | Stage III | T3 | M0 | N1 | low |
| TCGA-EI-6514 | 59 | FEMALE | Stage II | T3 | M0 | N0 | low |
| TCGA-EI-6881 | 60 | MALE | Stage III | T3 | M0 | N1 | low |
| TCGA-EI-6882 | 59 | MALE | Stage II | T3 | M0 | N0 | low |
| TCGA-EI-6883 | 63 | MALE | Stage II | T3 | M0 | N0 | high |
| TCGA-EI-6884 | 71 | MALE | Stage III | T3 | M0 | N1 | high |
| TCGA-EI-6885 | 57 | FEMALE | Stage IV | T3 | M1 | N1 | high |
| TCGA-EI-6917 | 33 | MALE | Stage III | T3 | M0 | N1 | high |
| TCGA-EI-7002 | 58 | MALE | Stage IV | T3 | M1 | N2 | high |
| TCGA-F4-6459 | 61 | FEMALE | Stage III | T3 | M0 | N2 | high |
| TCGA-F4-6460 | 51 | FEMALE | Stage III | T3 | M0 | N1 | high |
| TCGA-F4-6461 | 41 | FEMALE | Stage III | T4 | M0 | N2 | high |
| TCGA-F4-6463 | 51 | MALE | Stage II | T3 | M0 | N0 | high |
| TCGA-F4-6569 | 60 | MALE | Stage I | T2 | M0 | N0 | low |
| TCGA-F4-6570 | 78 | FEMALE | Stage II | T3 | M0 | N0 | low |
| TCGA-F4-6703 | 64 | MALE | Stage II | T3 | M0 | N0 | high |
| TCGA-F4-6704 | 60 | MALE | Stage III | T3 | unknow | N2 | high |
| TCGA-F4-6805 | 58 | FEMALE | Stage II | T3 | M0 | N0 | low |
| TCGA-F4-6806 | 59 | FEMALE | Stage I | T2 | M0 | N0 | low |
| TCGA-F4-6807 | 51 | FEMALE | Stage III | T3 | M0 | N2 | low |
| TCGA-F4-6808 | 54 | FEMALE | Stage I | T1 | M0 | N0 | low |
| TCGA-F4-6809 | 52 | FEMALE | Stage IV | T3 | M1 | N1 | high |
| TCGA-F4-6854 | 77 | FEMALE | Stage II | T3 | M0 | N0 | low |
| TCGA-F4-6855 | 70 | FEMALE | Stage II | T3 | M0 | N0 | high |
| TCGA-F4-6856 | 45 | MALE | Stage I | T2 | M0 | N0 | low |
| TCGA-F5-6571 | 62 | FEMALE | Stage II | T3 | M0 | N0 | low |
| TCGA-F5-6814 | 57 | MALE | Stage II | T3 | M0 | N0 | high |
| TCGA-F5-6861 | 60 | FEMALE | Stage II | T3 | M0 | N0 | low |
| TCGA-F5-6863 | 71 | FEMALE | Stage III | T4 | M0 | N1 | high |
| TCGA-F5-6864 | 74 | FEMALE | Stage III | T3 | M0 | N2 | high |
| TCGA-G4-6293 | 49 | FEMALE | Stage III | T3 | M0 | N1 | low |
| TCGA-G4-6294 | 75 | MALE | Stage IV | T3 | M1 | N1 | low |
| TCGA-G4-6295 | 70 | FEMALE | Stage II | T3 | M0 | N0 | high |
| TCGA-G4-6297 | 55 | FEMALE | Stage IV | T3 | M1 | N2 | high |
| TCGA-G4-6298 | 90 | MALE | Stage III | T4 | unknow | N1 | high |
| TCGA-G4-6299 | 69 | MALE | Stage III | T3 | M0 | N2 | high |
| TCGA-G4-6302 | 90 | FEMALE | Stage II | T3 | M0 | N0 | high |
| TCGA-G4-6303 | 54 | FEMALE | Stage IV | T3 | M1 | N1 | high |
| TCGA-G4-6304 | 66 | FEMALE | Stage II | T4 | M0 | N0 | high |
| TCGA-G4-6306 | 71 | MALE | unknow | T2 | M0 | N0 | low |
| TCGA-G4-6307 | 37 | FEMALE | Stage III | T3 | M0 | N1 | low |
| TCGA-G4-6309 | 40 | FEMALE | Stage III | T3 | M0 | N1 | low |
| TCGA-G4-6310 | 69 | MALE | Stage III | T3 | M0 | N1 | high |
| TCGA-G4-6311 | 80 | MALE | Stage III | T3 | unknow | N1 | high |
| TCGA-G4-6314 | 76 | FEMALE | Stage IV | T3 | M1 | N2 | high |
| TCGA-G4-6315 | 66 | MALE | Stage IV | T3 | M1 | N1 | low |
| TCGA-G4-6317 | 51 | FEMALE | Stage III | T3 | unknow | N2 | high |
| TCGA-G4-6320 | 73 | MALE | Stage III | T3 | unknow | N1 | low |
| TCGA-G4-6321 | 60 | FEMALE | Stage III | T2 | unknow | N1 | low |
| TCGA-G4-6322 | 65 | MALE | Stage III | T3 | unknow | N1 | high |
| TCGA-G4-6323 | 50 | MALE | Stage I | T1 | unknow | N0 | low |
| TCGA-G4-6586 | 73 | FEMALE | Stage II | T3 | M0 | N0 | low |
| TCGA-G4-6588 | 58 | FEMALE | Stage II | T3 | M0 | N0 | low |
| TCGA-G4-6625 | 77 | FEMALE | Stage II | T3 | M0 | N0 | low |
| TCGA-G4-6626 | 90 | MALE | Stage II | T3 | M0 | N0 | low |
| TCGA-G4-6627 | 84 | MALE | Stage II | T3 | M0 | N0 | low |
| TCGA-G4-6628 | 78 | MALE | Stage I | T2 | M0 | N0 | low |
| TCGA-G5-6233 | 74 | MALE | unknow | T3 | M0 | N2 | high |
| TCGA-NH-A50T | 68 | FEMALE | Stage II | T3 | unknow | N0 | low |
| TCGA-NH-A50U | 42 | MALE | Stage IV | T4 | M1 | N0 | high |
| TCGA-NH-A50V | 69 | MALE | Stage III | T3 | M0 | N2 | high |
| TCGA-NH-A5IV | 90 | FEMALE | Stage II | T3 | unknow | N0 | high |
| TCGA-NH-A6GA | 58 | MALE | Stage III | T4 | unknow | N2 | high |
| TCGA-NH-A6GB | 71 | FEMALE | Stage III | T3 | unknow | N2 | high |
| TCGA-NH-A6GC | 66 | FEMALE | Stage IV | T4 | M1 | N1 | high |
| TCGA-NH-A8F7 | 53 | FEMALE | Stage II | T3 | unknow | N0 | low |
| TCGA-NH-A8F8 | 79 | MALE | Stage IV | T4 | M1 | N2 | high |
| TCGA-QG-A5YV | 64 | FEMALE | Stage III | T4 | unknow | N1 | high |
| TCGA-QG-A5YW | 55 | FEMALE | Stage III | T3 | unknow | N2 | high |
| TCGA-QG-A5YX | 61 | FEMALE | Stage II | T3 | unknow | N0 | low |
| TCGA-QG-A5Z2 | 61 | MALE | Stage I | T2 | M0 | N0 | high |
| TCGA-QL-A97D | 84 | FEMALE | Stage I | T2 | unknow | N0 | low |
| TCGA-RU-A8FL | 51 | MALE | Stage III | T3 | unknow | N2 | high |
| TCGA-SS-A7HO | 44 | FEMALE | Stage II | T4 | M0 | N0 | low |
| TCGA-T9-A92H | 82 | MALE | Stage II | T3 | M0 | N0 | low |
| TCGA-WS-AB45 | 52 | FEMALE | Stage II | T3 | unknow | N0 | high |
